# Temporal variations of ambient air pollutants and meteorological influences on their concentrations in Tehran during 2012–2017

**DOI:** 10.1038/s41598-019-56578-6

**Published:** 2020-01-15

**Authors:** Fatemeh Yousefian, Sasan Faridi, Faramarz Azimi, Mina Aghaei, Mansour Shamsipour, Kamyar Yaghmaeian, Mohammad Sadegh Hassanvand

**Affiliations:** 1 Department of Environmental Health Engineering, School of Public Health, Tehran University of Medical Sciences, Tehran, Iran; 2 Center for Air Pollution Research (CAPR), Institute for Environmental Research (IER), Tehran University of Medical Sciences, Tehran, Iran; 3 Nutrition Health Research Centre, Department of Environment Health, School of Health and Nutrition, Lorestan University of Medical Sciences, Khorramabad, Iran; 4 Department of Research Methodology and Data Analysis, Institute for Environmental Research, Tehran University of Medical Sciences, Tehran, Iran

**Keywords:** Earth and environmental sciences, Earth and environmental sciences, Earth and environmental sciences, Atmospheric science, Atmospheric science

## Abstract

We investigated temporal variations of ambient air pollutants and the influences of meteorological parameters on their concentrations using a robust method; convergent cross mapping; in Tehran (2012–2017). Tehran citizens were consistently exposed to annual PM_2.5_, PM_10_ and NO_2_ approximately 3.0–4.5, 3.5–4.5 and 1.5–2.5 times higher than the World Health Organization air quality guideline levels during the period. Except for O_3_, all air pollutants demonstrated the lowest and highest concentrations in summertime and wintertime, respectively. The highest O_3_ concentrations were found on weekend (weekend effect), whereas other ambient air pollutants had statistically significant (P < 0.05) daily variations in which higher concentrations were observed on weekdays compared to weekend (holiday effect). Hourly O_3_ concentration reached its peak at 3.00 p.m., though other air pollutants displayed two peaks; morning and late night. Approximately 45% to 65% of AQI values were in the subcategory of unhealthy for sensitive groups and PM_2.5_ was the responsible air pollutant in Tehran. Amongst meteorological factors, temperature was the key influencing factor for PM_2.5_ and PM_10_ concentrations, while nebulosity and solar radiation exerted major influences on ambient SO_2_ and O_3_ concentrations. Additionally, there is a moderate coupling between wind speed and NO_2_ and CO concentrations.

## Introduction

The constructed Global Exposure Mortality Model by Burnett *et al*. (2018) estimated that exposure to ambient air pollution in 2015 was approximately responsible for nine million premature deaths globally^1^. Ambient air pollution exposure-related health effects mainly occurred in megacities of developing countries because of high ambient air pollutant concentrations^2^. Tehran as the capital and most populous city of Iran has faced intense ambient air pollution, particularly criteria air pollutants (PM_10_, PM_2.5_, O_3_, NO_2_, SO_2_ and CO), in the last two decades due to unsustainable development of industrialization and urbanization, the ever-growing automotive fleet and their emissions alongside ineffective national ambient air quality standards and Middle Eastern dust storm^3–6^. In fact, ambient air pollution in Tehran has become one of the most challenging environmental issues for Iranian central government, authorities, policy-makers, Tehran citizens, national and international researchers^3,7–9^. It is estimated that approximately 98% of CO, 75% of PM_2.5_ and 46% of NO_X_ are emitted from mobile sources in Tehran^4,10^, confirming the need for appropriate sustainable control policies and regulations against vehicular traffic, such as mandatory applying state-of-the-art technologies to reduce road traffic-related emissions, and more effective and serious implementation of transportation policies^4^. Also, energy conversion (e.g. power plants and oil refineries) is responsible for 25% of NO_X_ and 20% of particulate matter emissions^11^. Approximately 23% of NO_X_ originated from the household and commercial sectors^11^. Furthermore, SO_2_ is the only ambient air pollutant dominated by emissions from industrial activities (about 22%), power plants and oil refineries (68%), while the rest of SO_2_ emissions comes from mobile sources^10^. In urban areas, ambient O_3_ is generated via a series of complex photochemical reactions involving solar radiation (SR) and O_3_-precursors, e.g., NO_X_, CO, reactive volatile organic compounds (VOCs) and methane^12^. Similar to other O_3_-precursors (NO_X_ and CO) in Tehran, VOCs are mainly emitted from mobile sources (approximately 86%)^4,13^. To date, numerous investigations have been conducted in Tehran that have focused on various issues of ambient air pollution, including investigation of chemical characterization of ambient particulate matter^14,15^ and their toxicological effects^16^, ambient particulate matter source apportionment^10^, health effects of ambient air pollutants^3,17^ and emission inventory of ambient air pollutants^7^. Although remarkable investigations have been conducted, a comprehensive and in-depth understanding temporal variability of all criteria air pollutants as well as the meteorological influences on their concentrations using a robust method remains unclear in Tehran to date. Firstly, previous investigations mainly considered temporal variability of one or two ambient air pollutants and their correlations with meteorological parameters (MPs) using Pearson or Spearman correlation analysis. Secondly, since various MPs interact closely with each other, the commonly used Pearson or Spearman correlation analysis may lead to biased findings^18^. Advanced approaches, including the convergent cross mapping (CCM) method and Granger causality test, instead of a simple correlation analysis, should be comprehensively utilized to quantify the influence of MPs on ambient air pollutant concentrations^19^. Finally, exploring temporal variations of all criteria air pollutants with proper approaches and their casual relationships with MPs are crucial to reveal whether the implemented air pollution control measures were successful or not, as well as can be used as a beneficial tool for air quality policy- and decision-makers to modify and revise air pollution controls strategies in order to more mitigate air pollutant concentrations and their health outcomes^20,21^. Therefore, this study was designed to investigate (**1)** temporal variations (annual, seasonal, monthly, daily and hourly) of criteria air pollutants; PM_2.5_, PM_10_, NO_2_, O_3_, SO_2_, and CO; as well as the long-term trend of air quality index (AQI) and (**2)** the influence of MPs such as temperature, precipitation, wind speed (WS), SR, relative humidity (RH), and nebulosity on the concentrations of ambient air pollutants in Tehran during the study period from 2012 to 2017.

## Results and Discussion

### Overview and annual trends of criteria air pollutant concentrations

**Figure 1:**
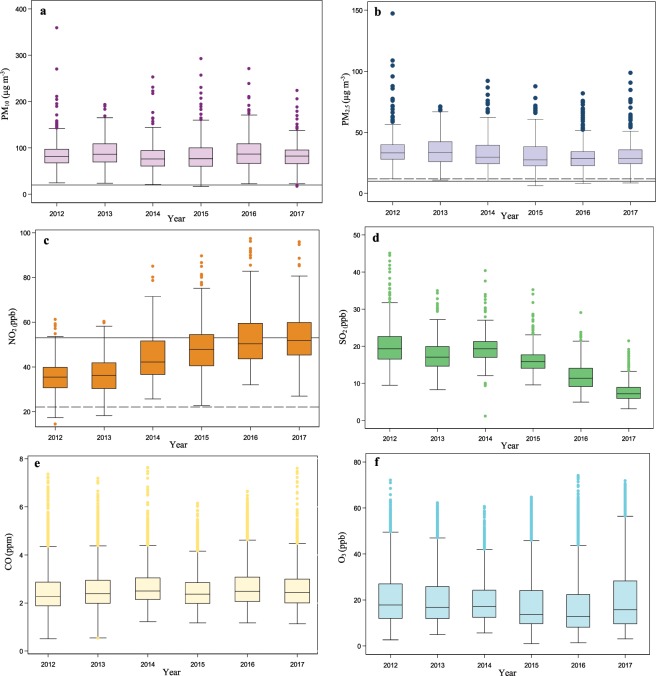
The year-boxplot of PM_10_ (**a**), PM_2.5_ (**b**), NO_2_ (**c**), and SO_2_ (**d**) based on 24-hr concentrations and CO (**e**) and O_3_ (**f**) based on 8-hr concentrations in Tehran from 2012 to 2017. Black solid lines and black long-dash lines represent the World Health Organization air quality guideline and Iranian standard levels, respectively.

**Figure** **1(a–f)** and **Table** **S1** compare the annual mean concentrations of six criteria air pollutants in Tehran during the study period from 2012 to 2017. The highest annual PM_2.5_, O_3_, and SO_2_ mean concentration was recorded in 2012 with approximately 36.0 μg m^−3^, 20.7 and 20.4 ppb, whereas the highest annual mean concentration of PM_10_ (90.0 μg m^−3^), NO_2_ (53.3 ppb) and CO (2.7 ppm) was found in 2013, 2017 and 2014, respectively. Also, the lowest annual mean concentration of PM_2.5_ and O_3_, NO_2_ and CO, PM_10_, and SO_2_ was observed in 2016, 2012, 2014, and 2017 with approximately 30.0 μg m^−3^ and 17.5 ppb, 35.6 ppb and 2.5 ppm, 79 μg m^−3^, and 7.9 ppb, respectively. Unfortunately, annual PM_10_, PM_2.5_, and NO_2_ mean concentrations were higher than the World Health Organization air quality guideline levels (20 and 10 μg m^−3^ for PM_10_ and PM_2.5_, and 22 ppb for NO_2_) during the entire study period **(Figure** **1a–c****)**.

A glance at the **Figure** **1a** provided reveals that the annual mean PM_10_ concentrations fluctuated between approximately 78.9 and 89.9 μg m^−3^ and had no a constant downward or upward trend over the entire study period (2012–2017). The non-parametric Mann-Kendall trend test and Sen’s slope estimator (MKTT-SSE) confirmed these findings **(Table** **1****)**. Compared to PM_10_, annual averages of ambient PM_2.5_ decreased from about 36.0 μg m^−3^ in 2012 to 31.5 μg m^−3^ in 2017; a decline of 14.3 percent from 2012 to 2017. On the other hand, according to MKTT-SSE, annual PM_2.5_ declined significantly (P < 0.05) with the slope of 1.2 μg m^−3^ per year **(Table** **1****)**. During the period from 2012 to 2017, among all ambient gaseous air pollutants, only annual mean concentration of NO_2_ and SO_2_ revealed a constant upward and downward trend, respectively **(Figure** **1c,d** and **Table** **1****)**. Annual mean SO_2_ concentrations declined from approximately 20.4 to 7.9 ppb between 2012 and 2017; a sharp fall of 61.5 percent **(Figure** **1d****)**. Regarding MKTT-SSE, SO_2_ decreased statistically significant (P < 0.05) with the slope of 2.5 ppb per year **(Table** **1****)**. By contrast, annual NO_2_ displayed a considerable upward trend in which its annual mean concentrations increased to approximately 53.3 ppb in 2017; an overall increase of 33 percent between 2012 and 2017. Based on MKTT-SSE, NO_2_ increased significantly (P < 0.05) with the slope of 4.2 ppb per year **(Table** **1****)**. For CO **(Figure** **1e****)**, annual mean concentrations fluctuated at somewhere between 2.5 and 2.7 ppm and showed a statistically non-significant M-shaped pattern **(Table** **1****)**. Similar to PM_10_ and CO, ambient O_3_ had a statistically non-significant fluctuating trend during the study period from 2012 to 2017 **(Figure**. **1f** and **Table** **1****)**. In summary, the main reasons behind the declining trend of annual PM_2.5_ and SO_2_ may be associated with various local air pollution control policies such as implementation of rules on emission standards and fuel quality enhancement (e.g. low sulfur diesel), phasing out of old/carburetor equipped vehicles, the mandatory use of diesel particulate filter, vehicle catalyst replacement, adopting EURO norms, conversion of diesel engines to compressed natural gas, as well as the extending public transportation, particularly subway and bus rapid transit^7,22,23^. As reported by Tehran Air Quality Control Company (TAQCC), during the period 2014–2017, the sulfur content of gasoline and diesel distributed in Tehran megacity was decreased from 200 and 7000 ppm to approximately 20 and less than 50 ppm, respectively^24^. The rising annual trend of NO_2_ as one of the most important road traffic emissions can be related to the increase of total number of vehicles in Tehran over time, because the number of registered vehicles in Tehran increased from around 3.8 million in 2012 to approximately 4.5 million in 2015^7^. Additionally, in the last decade, Tehran megacity has witnessed construction of approximately 31 km of highways annually^25^.

**Table 1:** MKTT-SSE for annual mean concentration of ambient air pollutants in Tehran from 2012 to 2017.

Air pollutants	Mann-Kendall trend (z, s)	Sen’s slope estimate (concentration^†^/year)
PM_10_	−1	−0.42
PM_2.5_	−11^*^	−1.17
NO_2_	15^*^	4.2
O_3_	−7	−0.62
SO_2_	−13^*^	−2.51
CO	5	0.02

(*P < 0.05, ^†^concentration: μg m^−3^ for PM_10_ and PM_2.5_, ppb for O_3_, NO_2_ and SO_2_, and ppm for CO).

### Seasonal and monthly patterns of ambient air pollutants

**Figure 2:**
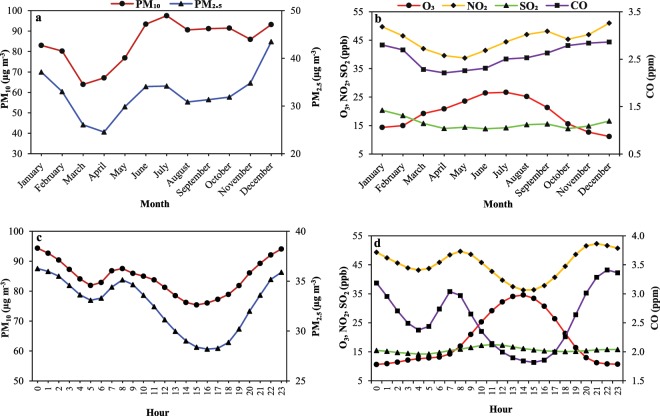
Temporal variations of ambient PM_10_, PM_2.5_ (**a**,**c**) and NO_2_, O_3_, SO_2_, CO (**b**,**d**) concentrations in Tehran during the study period (2012–2017).

To better reveal the most polluted seasons and months in Tehran, seasonal and monthly concentrations of all ambient air pollutants were investigated during the period 2012–2017. Based on the Iranian calendar, four seasonal periods were examined as following: spring (21 March to 21 June), summer (22 June to 22 September), fall (23 September to 21 December), and winter (22 December to 20 March). According to regression model analysis (RMA) for seasonal and monthly variations, all ambient air pollutants showed statistically remarkable seasonal and monthly variations **(Figure** **S1**(a,b), Figure 2(a,b) and **Tables** **S2** to **S8****)**. Ambient PM_2.5_, NO_2_, SO_2_ and CO revealed significantly higher mean concentrations during colder seasons and months, whereas PM_10_ showed the highest values during summertime (93.2 μg m^−3^), followed by fall (88.4 μg m^−3^), winter (83.4 μg m^−3^), and spring (73.4 μg m^−3^) **(Figure** **S1a****)**. The mean concentrations of ambient PM_2.5_ (μg m^−3^), NO_2_ (ppb) and SO_2_ (ppb) were in the order of winter (36.2, 48.1, and 18.7) > fall (34.6, 46.8, and 14.9) > summer (32.5, 46.1, and 14.7) > spring (27.6, 39.4, and 14.2) **(Figure** **S1a,b****)**. In terms of CO, the highest concentrations were found during fall (2.8 ppm) and wintertime (2.7 ppm), whereas the lowest concentrations were observed during spring (2.2 ppm) and summertime (2.5 ppm). Compared to other gaseous air pollutants, O_3_ displayed the highest concentrations during the summer and spring months, especially July (26.7 ppb), when SR **(Figure** **S2a****)**, temperature **(Figure** **S2a****)**, hydroxyl radical as the most important oxidant species for the formation of O_3_, VOCs and photochemical reactions are higher^26,27^. Observed seasonal and monthly patterns for PM_2.5_, NO_2_, SO_2_ and CO can be attributed to a combination of unfavorable meteorological conditions, including stagnant weather, reduced horizontal and vertical WS, higher nebulosity, reduced sunshine time, lower SR, temperature inversion and lower the boundary layer during the coldest seasons and months compared to summer and spring months **(Figure** **S2****)**^28–31^. In relation to PM_10_, exactly similar to Ahvaz city, Middle East dust storm was responsible for the peak concentration of PM_10_ during summer in Tehran^11,32–35^. High concentrations of PM_10_ during fall and winter months are more likely due to the aforementioned reasons for PM_2.5_, NO_2_, SO_2_ and CO concentrations. Our results, particularly seasonal and monthly variations of gaseous air pollutants, are consistent with the findings reported by S. Squizzato and colleagues (2018) across New York State and the results of R. Li and colleagues (2017) in 187 Chinese cities^26,29^.

Nowruz Persian New Year holidays which late approximately 2 weeks from late March to early April each year are the most notable and the longest holiday in Iran^4,36^. Consequently, vehicular traffics as the most important emission source of ambient air pollutants, particularly PM_2.5_ and NO_2_, reduce considerably in Tehran during this period^3,4^. As expected, during the Nowruz holidays, the concentrations of air pollutants were significantly (P < 0.05) declined compared to the rest of year **(Table** **S9****)**. Moreover, the mean PM_10_, PM_2.5_ and NO_2_ concentrations as the most notable marker of road traffic emissions were 41.0 μg m^−3^, 17.0 μg m^−3^ and 37 ppb during the Nowruz holidays, whereas their concentrations over the rest of year were 86 μg m^−3^, 33 μg m^−3^ and 45 ppb, respectively **(Figure** **S3****)**.

### Daily and hourly variations of air pollutants

**Figures** **2(c,d)** and **S1(c,d)** illustrate hourly and daily variations of ambient air pollutants in Tehran during the study period from 2012 to 2017. A glance at the **Figure** **2c** and **Figure** **S1c** provided reveals that not only hourly variations of ambient PM_10_ and PM_2.5_ but also daily pattern of them are exactly similar. Here, we considered Saturday to Thursday as weekdays/working days and Friday as weekend^3^. In Tehran, by beginning of the working days, vehicle traffic and other emission sources significantly increase mainly due to rising activity of Tehran citizens and daily commuters from other cities of Iran, about 3.5 million commuters^24,25,37^. Therefore, daily mean concentrations of PM_2.5_ and PM_10_ start to increase and reach their peaks at approximately 33.9 and 88.6 μg m^−3^ on Wednesday, respectively. In fact, ambient PM_2.5_ and PM_10_ concentrations begin to accumulate on the atmosphere over weekdays and they reach their maximum concentrations on Wednesdays. Not surprisingly, the lowest daily mean concentration of ambient PM_2.5_ and PM_10_ was recorded on Fridays with approximately 30.3 and 76.8 μg m^−3^, which is known as the “holiday effect”, followed by Saturdays with 31.8 and 82.5 μg m^−3^. Our results are consistent with the findings of Faridi *et al*., and O. Alizadeh-Choobari *et al*. in Tehran megacity and the results of Maleki and colleagues in Ahvaz city^3,35,36^. It is interesting to note that the daily pattern of PM_10_ and PM_2.5_ in our study is exactly similar to day-to-day variations of vehicle traffic in Tehran reported by S. A. H. hassanpour Matikolaei *et al*.^25^. In addition to the above-mentioned reason, the decrease of ambient air pollution on Saturdays can be related to the self-purification capacity of atmosphere on weekend. According to RMA, daily PM_2.5_ and PM_10_ on working days was statistically significant higher compared to weekends during the period from 2012 to 2017 **(Tables** **S10** and **S11****)**. In terms of hourly variation of ambient PM_2.5_ and PM_10_, we observed two peaks; one in the morning (8:00) and another in the late night (00:00) **(Figure** **2c**, **Tables** **S12** and **S13****)**. The morning peaks with 35.1 and 87.5 μg m^−3^ for PM_2.5_ and PM_10_ were significantly smaller compared to the peaks observed in the late night **(Figure** **2c****)**. The morning peak is only likely due to vehicular traffic in the morning, whereas another peak can be related to the traffic of light-duty vehicles in the late afternoon and early evening accompanied by increasing heavy-duty vehicles-related traffic during nighttime (after 22:00) as a traffic restriction, construction/demolition activities and their related-waste transfer and management, open burning of solid waste, switching off the air pollution control equipment at night, secondary particles formation, as well as decreasing boundary layer height^3,8,29,36^. Moreover, as shown in **Figure** **2c**, two valleys are obviously visible for hourly PM_2.5_ and PM_10_ in the early morning (from 4:00 to 6:00) and from mid-morning to late afternoon/early evening. The latter valley is most likely owing to increasing boundary layer depth together with reduced traffic-related emissions and the increase of WS^38–40^. Generally speaking, based on RMA **(Tables** **S14** and **Figure** **S4****)**, the nighttime (from 21:00 to 7:00) concentrations of PM_2.5_ and PM_10_ were significantly higher in comparison to the daytime (between 8:00 and 20:00) concentrations, which can be explained by the above-mentioned reasons^41–43^. Finally, it is worth noting that hourly patterns of PM_2.5_; as a notable marker of combustion emissions from road traffic; and PM_10_ in Tehran are similar to hourly traffic-related emissions, to be exact^11^. As shown in **Figure** **S1d**, daily mean concentrations of NO_2_, O_3_, SO_2_ and CO were about constant around a value from Saturday to Thursday, followed by a statistically slight increase in mean concentration of O_3_ and a statistically small reduction in mean concentrations of NO_2_, SO_2_ and CO on weekend. These slight decreases and rises of ambient gaseous air pollutants on Friday in comparison to other days of week were statistically significant based on the results of RMA **(Tables** **S15** to **S18****)**. The decrease of NO_2_, SO_2_ and CO concentrations at the end of week can be mainly attributed to lower vehicle traffic compared to the other days of week, whereas the increase of O_3_ as a secondary air pollutant is most likely owing to decreasing O_3_ destruction by the reduced titration effect of NO_X_ and other ambient air pollutant precursors on weekend **(Tables** **S15** to **S18****)**^44–46^. In reality, similar to PM_2.5_, NO_2_ is used as an important marker for combustion emissions, especially from road traffic and its decrease on weekend represents the reduction of traffic^40^. Similar to ambient PM_10_ and PM_2.5_, hourly variation of NO_2_ and CO clearly exhibited two peaks and two valleys, mainly reflecting the effect of traffic emissions and meteorological conditions on CO and NO_2_ during a day^24,37,44,47,48^. After the observed peaks at 7:00 and 8:00, the concentrations of CO and NO_2_ started to decrease and reached their lowest concentrations at 14:00 and 15:00 due to a combination of increasing boundary layer height, WS, SR and photochemical reactions in order to produce O_3_ coupled with decreasing vehicle traffic emissions as evident by decreased ambient NO_2_^29,45,49^. Based on RMA, similar to PM_10_ and PM_2.5_, the nighttime concentrations of NO_2_ and CO were statistically significantly higher than those observed during the daytime, mainly because of the above-mentioned reasons, as well as the lack of photochemical reactions for their destruction and consumption to produce ambient O_3_ **(Tables** **S19** to **21****)**^45,50–52^. Furthermore, hourly O_3_ revealed a sharp mountain-peak-shaped pattern after midday (14:00) owing to higher SR and photochemical reactions in the early afternoon^3^. Unlike other air pollutants, SO_2_ revealed no specific hourly pattern, though its hourly variation was statistically significant in the vast majority of hours **(Table** **S22****)**. According to RMA, unlike PM_2.5_, PM_10_, NO_2_ and CO, the daytime concentration of SO_2_ was statistically higher than that during nighttime **(Figure** **S4****)**. In terms of the holiday/weekend effects on ambient air pollutant concentrations, our findings are consistent with the findings of Zhang, Y.-L. and Cao, F. (2015) across Yangtze River Delta, the Pearl River Delta and the Beijing-Tianjin-Hebei regions in China^53^, as well as the findings of S. Squizzato and colleagues (2018) across New York State^29^.

### AQI and responsible ambient air pollutant

**Figure 3:**
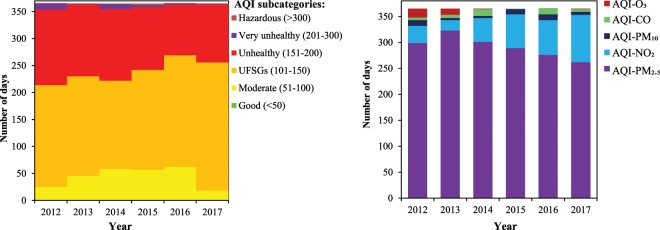
The AQI-subcategories’ figures (left) and contribution of each air pollutant in AQI values (right) in Tehran during the study period from 2012 to 2017.

**Figure** **3** reveals the subcategories of daily AQI values, as well as the contribution of each air pollutant in AQI figures in Tehran between 2012 and 2017. Daily AQI figures were in the range of 63–497 during the period from 2012 to 2017 **(Figure** **3** and **Table** **S23****)**. Furthermore, the highest daily AQI (497) was found in 2014, whereas the lowest value (63) was recorded in 2016. Unfortunately, we had no AQI value less than 50, as good subclass of AQI, in Tehran during the mentioned period **(Figure** **3** and **Table** **S24****)**. A glance at the **Figure** **3** provided shows that the number of unhealthy for sensitive groups’ (UFSGs) days had a V-shaped pattern over the whole study period, in which the number of days with the subcategory of UFSGs decreased from 189 to 164 days; a slight decrease of 25 days; during the first three years of the study (2012–2014). Afterwards, it increased considerably to 238 in 2017; an overall increase of 53 days. During the first five years (2012–2016) of the study, the number of days with moderate subcategory has more than doubled, from 25 days in 2012 to 62 days in 2016 **(Table** **S24****)**. The MKTT-SSE confirmed this increasing trend **(Table** **S25****)**. Fortunately, unhealthy days for Tehran citizens showed a significant decrease by 46 days between 2012 and 2016 **(Table** **S25****)**. The number of days with very unhealthy and hazardous conditions declined erratically over the entire study period. As can be noticed in **Figure** **3**, all ambient air pollutants, with the exception of SO_2_, led to decrease air quality status in Tehran during the study period 2012–2017. Moreover, ambient PM_2.5_ was the most frequent (from 262 to 323 days, approximately between 72.0% and 88.5% out of all days each year) major air pollutant in Tehran during the 6-year study from 2012 to 2017, followed by NO_2_ (20–91 days, approximately from 5% to 25% out of all days each year) as the second frequent major ambient air pollutant in Tehran. On the other hand, PM_2.5_ with 88.5% out of all days showed the highest contribution in daily AQI figures for 2013, whereas the lowest contribution for ambient PM_2.5_ with 72% out of all days was observed in the year 2017. Compared to PM_2.5_, the highest contribution for NO_2_ in daily AQI figures was recorded in 2017, whereas the lowest contribution of NO_2_ (5% out of all days) was found in 2013. Overall, CO and O_3_ had the lowest contributions in daily AQI in Tehran over the study period (2012–2017) **(Figure** **3****)**.

### The causality effect of individual MPs on six criteria air pollutants

**Figure 4:**
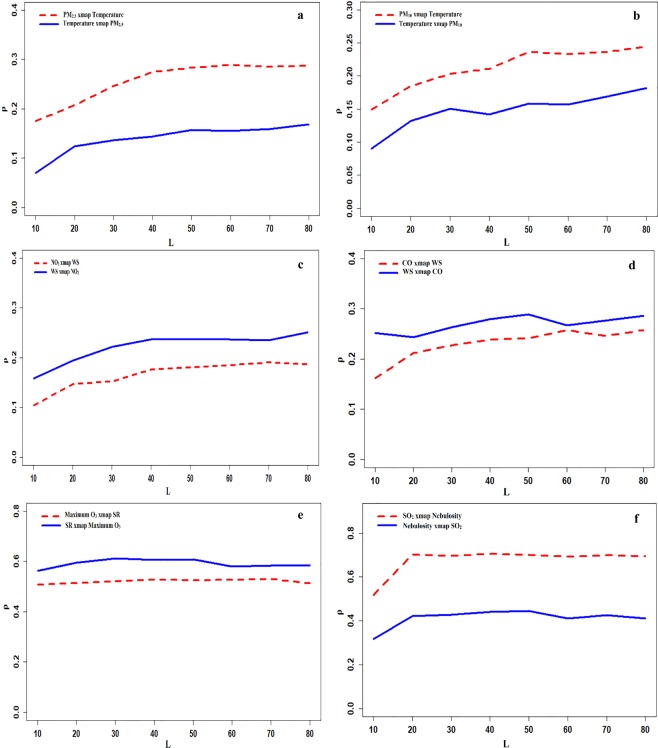
Exemplary CCM test results to show the causality between MPs and the concentrations of ambient PM_2.5_ (**a**), PM_10_ (**b**), NO_2_ (**c**), CO (**d**), O_3_ (**e**), and SO_2_ (**f**) in Tehran.

Herein, to avoid the influences from other probable factors and mirage correlations, we utilized a robust causality analysis approach; the CCM method; to extract the influences of different individual MP on ambient air pollutant concentrations. With a comprehensive understanding of interactions between all ambient air pollutants’ concentrations and MPs, this study can provide useful results in order to better predict and control ambient air pollution status in Tehran for policy-makers and environmental science researchers. Moreover, previously conducted studies^28,54^ indicated that MPs are one of the most notable factors causing variations of ambient air pollution over a city. Since it is not feasible to present all convergent maps, hereunder, we display six exemplary convergent maps to demonstrate the mechanism of the CCM method (Figure 4a–f). Hence, the rest of causality maps are presented in the supplementary file in detail **(Figures** **S5** to **S10****)**. Additionally, it should be noted that in the present study was explained the influences of MPs on ambient air pollutant concentrations and the influences of ambient air pollutant concentrations on MPs were not presented. Also, we examined the correlation analysis between air pollutant concentrations and MPs using Spearman correlation analysis **(Table** **S28****)** because the CCM analysis cannot show the direction of the influences of MPs on ambient particulate matter and gaseous air pollutants^18^. In fact, the positive/negative direction from Spearman correlation analysis provides a reliable reference for comprehensive understanding the mechanism how MPs influence ambient air pollutant concentrations^18^. Quantified causality of individual MPs on air pollutant concentrations by the CCM method; the ρ value; is a more reliable indicator and can remarkably differ a lot from the Spearman correlation coefficient; the r value. On the other hand, a large r value for a MP may correspond to a much smaller ρ value^20^. **Figure** **4(a–f)** illustrates the quantitative coupling between MPs and air pollutant concentrations by using the CCM method. As shown in **Figure** **4a**, there was a moderate bidirectional coupling between ambient PM_2.5_ concentrations and temperature (ρ value ~ 0.32). According to correlation coefficients **(Table** **S26****)**, temperature demonstrated a negative influence on ambient PM_2.5_ concentrations with r value equal to −0.124^20^. In reality, according to the correlation and CCM analysis (Table S26 and **Figure** **4a****)**, a negative bidirectional coupling between temperature and ambient PM_2.5_ concentrations was found in Tehran during the study period (2012–2017). Similar to PM_2.5_, a moderate bidirectional interaction was found between ambient PM_10_ concentrations and temperature with ρ value equal to 0.28. The results of the CCM analysis indicated that WS with a ρ value in the range of 0.20–0.25 had a weak influence on ambient NO_2_ and CO concentrations, as illustrated in **Figure** **a(c,d)**. Additionally, a statistically significantly (P < 0.05) negative correlation was found between WS and ambient NO_2_ (−0.28) and CO (−0.46) concentrations, as shown in **Table** **S28**. On the other hand, a negative bidirectional coupling between WS and the concentrations of ambient NO_2_ and CO was found based on the results of the CCM and Spearman correlation analysis. As expected, SR as the most notable influential MP displayed a moderate to strong influence (ρ value ~ 0.60) on ambient O_3_ concentration **(Figure** **4e****)**. In this case, based on Table S26, O3 had a high positive correlation with SR (0.55) and temperature (~0.63). Our findings were found that there was a positive bidirectional coupling between SR and ambient O_3_ concentrations in Tehran which was consistent with previous study in Beijing^18^. As **Figure** **4f** demonstrates strong coupling between SO_2_ concentrations and nebulosity (ρ value = 0.68) which is likely due to lower dispersions during temperature inversion and lower the boundary layer in coldest situations. As expected, ambient SO_2_ concentration had a statistically significantly (P < 0.05) negative correlation with RH (r value equal to −0.15), precipitation (−0.19) and nebulosity (−0.27) as markers of colder status **(Table** **S26****)**.

### Recommendations for air quality improvement in Tehran

In Tehran, major sources of criteria air pollutants, with the exception of O_3_ as a secondary air pollutant, have previously been reported arising from road traffic-related emissions (the highest contribution for CO, PM_2.5_ and NO_X_), industrial activities (as the important emission sources of SO_2_, PM and NO_X_), energy conversion sector (as another important contributor for NO_X_ and PM emissions and the most notable contributor for SO_2_), as well as household and commercial sectors (as the other contributors for NO_X_ emissions)^10,11,55^. Therefore, based on the successful short- and long-term programs in other megacities of developed and developing countries^24,56,57^, we recommend a policy mix in order to improve the air quality situation in Tehran megacity: (**1)** the heavy- and light-duty vehicles (HDVs and LDVs) replacement program via providing financial incentives to owners of old vehicles to trade them with new/less polluting ones; (**2**) expanding and improving public transportation (Bus-Raid Transport, Light Rail Transport and metro lines); (**3)** adopting higher fuel quality standards (Euro 5 and 6); (**4)** slashing fuel subsidies; (**5)** incentivizing electric and hybrid vehicles, including cars, motorcycles and HDVs; (**6)** incentivizing non-motorized transport such as walking or cycling; (**7)** stricter environmental taxes and penalties for industrial activities and energy conversion sectors (e.g., power plants and oil refineries); (**8)** utilizing sustainable energy technologies in industrial activities and energy conversion sectors and (**9)** implementation of green tax for household and commercial sectors.

### Limitations of this study

As mentioned below, the ambient air quality data were not obtained by the authors of the current work through their own research study rather the ambient air pollutants’ data were obtained from Tehran Air Quality Control Company (TAQCC) as a governmental organization that is responsible for ambient air quality monitoring in Tehran. Though we processed and cleaned ambient air quality data obtained from TAQCC, the authors have no information regarding the collocated operations of the instruments, flow calibration, and quality assurance and quality control (QA/QC) at the network level. Also, based on personal communication, the technical officer of air quality monitoring stations (AQMSs) mentioned that they follow QA/QC procedures exactly similar to the manual of monitoring instruments used at each AQMS.

## Methods

### Air quality and meteorological data

Real-time hourly air quality data (PM_2.5_, PM_10_, NO_2_, O_3_, SO_2_ and CO) in Tehran between 2012 and 2017 from twenty-one active AQMSs which belong to TAQCC were obtained from the website of http://airnow.tehran.ir/home/DataArchive.aspx. At all AQMSs, ambient PM_2.5_ and PM_10_, O_3_, NO_2_, SO_2_, and CO are monitored using the beta-attenuation (Met One BAM-1020, USA; and Environment SA, MP 101 M, France), UV-spectrophotometry (Ecotech Serinus 10 Ozone Analyzer, Australia), chemiluminescence (Ecotech Serinus 40 Oxides of Nitrogen Analyzer, Australia), ultraviolet fluorescence (Ecotech Serinus 50 SO_2_ Analyzer, Australia), and non-dispersive infrared absorption (Ecotech Serinus 30 carbon monoxide Analyzer, Australia) methods, respectively (based on personal communication with technical officer of AQMSs from TAQCC). Additionally, the organization follows the QA/QC procedures exactly similar to the manual of monitoring instruments used at each AQMS. For gaseous air pollutants, the instruments are automatically calibrated/checked every 7 days for span and zero calibration. Multipoint calibrations are manually performed approximately every six months according to the manual of monitoring instruments. Additionally, gas analyzers are calibrated following relocation, after any repair or service that might affect their calibration, following an interruption in operation of more than a few days, upon any indication of analyzer malfunction. For ambient PM monitoring instruments, the routine QC and maintenance procedure (nozzle, vane and the PM inlet cleaning, very sharp cut cyclone particle size separator cleaning, leak checking, temperature/pressure/flow calibration) are performed monthly with the exception of the filter tape change, which mainly takes place bi-monthly. Furthermore, additional maintenance steps (the pump muffler cleaning/replacing, the 72-hour zero test and the membrane span foil checking, etc.) are performed every 6 months and every 12 months. **Figure** **S11** shows the spatial distribution of AQMSs. Furthermore, detailed information regarding AQMSs is provided in **Table** **S27**. Additionally, meteorological data such as temperature, WS, SR, nebulosity, precipitation, and RH were derived from Tehran Province Metrological Administration. **Table** **S28** illustrates descriptive statistics of meteorological data during entire study period (2012–2017).

### Air quality data processing

Prior to analyzing hourly air pollutant concentration for the mentioned objectives earlier, air quality data processing and cleaning were conducted on only AQMSs with hourly data coverage more than 70% according to Z-score method in order to check and remove outlier hourly data from original hourly time series datasets^3,21,58^. Hourly air quality data were transformed into Z-score and outlier data removed from the subsequent computation according to the following conditions: (**1**) having an absolute Z-score larger than 4 (|Z_t_ | > 4), (**2**) the increment from the previous hourly value being larger than 9 (Z_t_ − Z_t−1_ > 9) and (**3**) the ratio of the hourly value to its centered rolling average of order 3 (RA3) being larger than 2 (Z_t_/RA3(Z_t_) > 2). The cleaned and processed hourly air quality data were used to account the averages of 1-hr, the running 8-hr and the 24-hr. Hourly concentrations at city-wide were computed according to the hourly data across all included AQMSs for each hour. Then, the running 8-hr average of O_3_ and the 24-hr average of other air pollutants were calculated for city.

### AQI and responsible ambient air pollutant in Tehran

To inform the general public regarding air quality status and its associated health risks, AQI as a daily index is a popular method of air quality knowledge translation^59,60^. This dimensionless index is divided into six subcategories with specified colors as following **(Table** **S29****)**: good (less than 50, green), moderate (51–100, yellow), UFSGs (101–150, orange), unhealthy (151–200, red), very unhealthy (201–300, purple), and hazardous (more than 300, maroon). In AQI approach, a daily ‘responsible air pollutant’ is identified for city to determine which criteria air pollutant contributes the most to the air quality status degradation. In this work, based on the breakpoints’ levels suggested by the U.S. EPA **(Table** **S29****)**, in order to calculate the AQI for PM_2.5_, PM_10_, O_3_ and CO, 24-hr average concentrations of PM_2.5_ and PM_10_ and 8-hr average concentrations of O_3_ and CO were computed from their hourly concentrations. Also, to calculate the AQI related to NO_2_ and SO_2_, their hourly concentrations were used. Next, amongst all AQI figures computed for six criteria air pollutants at all AQMSs, the highest AQI was finally considered as the daily AQI and responsible air pollutant for city. The following Eq. (1) was used to compute AQI for each air pollutant^59^.

1 $${{\rm{AQI}}}_{{\rm{ap}}}=\frac{{{\rm{AQI}}}_{{\rm{uc}}}-{{\rm{AQI}}}_{{\rm{lc}}}}{{{\rm{BP}}}_{{\rm{uc}}}-{{\rm{BP}}}_{{\rm{lc}}}}({{\rm{C}}}_{{\rm{ap}}}-{{\rm{BP}}}_{{\rm{lc}}})+{{\rm{AQI}}}_{{\rm{lc}}}$$

where, AQI_ap_ represents the index for given air pollutant (ap); AQI_uc_ and AQI_lc_ represent the index values corresponding to upper and lower of each breakpoint category (BP), respectively; C_ap_ is the concentration of each air pollutant; BP_uc_ and BP_lc_ are the upper and lower concentrations of air pollutant at each breakpoint category, respectively.

### Statistical analysis

#### Temporal characteristics

In order to reveal upward and downward trends (annual mean concentrations of each air pollutant and AQI), their magnitude, as well as whether their magnitude were statistically significant (P < 0.05) or not, the non-parametric MKTT-SSE was run^27,38^. RMA with dummy variables was run to illustrate the differences of mean concentrations at hours, days, months, and seasons for each air pollutant^12^. Similarly, the differences between nighttime and daytime concentrations, as well as the effect of Nowruz holidays on the concentrations of ambient air pollutants compared to the rest of year were assessed using RMA with dummy variables^12^. The mentioned analyses were conducted using State software.

#### Quantifying the causality influences of MPs on ambient air pollutant concentrations

Due to complicated interactions between MPs and ambient air pollutant concentrations in the atmospheric environment, in fact, it is highly difficult to quantify the causality of MPs on ambient air pollutants through simple Pearson and Spearman correlation analyses^18^. Instead, a robust approach for quantitative causality analysis is proposed by previous studies^18,19^. The CCM method is suitable for detecting causation in time-series data^18^. In this method, by examining the temporal changes of two time-series datasets, their bidirectional coupling can effectively be featured with a convergent map^18^. Furthermore, the CCM approach detects effectively even weak to moderate coupling in time-series variable. If the influence of one variable on another variable is indicated using a convergent curve with rising time series length, then the causality is detected. On the other hand, a curve without any convergence demonstrates no causality between the two variables^19^. The predictive skill (defined as the ρ value), ranging from 0 to 1, shows the strength of influences from one variable on the other^19,20^. This approach cannot show the direction of the influence of MPs on air pollutant concentrations. Therefore, we investigated the positive/negative direction of their influences on air pollutant concentrations using Spearman correlation analysis^20^. To depict the convergent maps of bidirectional causal relationships, the rEDM package in R software version 3.4.5 was used.

## Supplementary information

Supplementary information
