# Supplementary material for: Temporal variations of ambient air pollutants and meteorological influences on their concentrations in Tehran during 2012–2017
Source: Sci Rep. 2020 Jan 15;10:292. doi: 10.1038/s41598-019-56578-6 (PMC6962207; doi:10.1038/s41598-019-56578-6)
Supplement: Supplementary file 1 — Supplementary information [file 41598_2019_56578_MOESM1_ESM.pdf]

# Temporal variations of ambient air pollutants and meteorological influences on their concentrations in Tehran during 2012-2017

**Fatemeh Yousefian [a, b], Sasan Faridi [a, b], Faramarz Azimi [c], Mina Aghaei [a], Mansour Shamsipour [d], Kamyar Yaghmaeian [a*], Mohammad Sadegh Hassanvand [a, b*]**

[a] Department of Environmental Health Engineering, School of Public Health, Tehran University of Medical Sciences, Tehran, Iran.
[b] Center for Air Pollution Research (CAPR), Institute for Environmental Research (IER), Tehran University of Medical Sciences, Tehran, Iran.
[c] Nutrition Health Research Centre, Department of Environment Health, School of Health and Nutrition, Lorestan University of Medical Sciences, Khorramabad, Iran
[d] Department of Research Methodology and Data Analysis, Institute for Environmental Research, Tehran University of Medical Sciences, Tehran, Iran.

[*] Corresponding authors:
K. Yaghmaeian, Department of Environmental Health Engineering, School of Public Health, Tehran University of Medical Sciences, Tehran, Iran. Telephone: +982188950188. E-mail: KYaghmaeian@gmail.com.
M.S. Hassanvand, 8th Floor, No. 1547, North Kargar Avenue, Tehran, Iran. Telephone: +982188978395. E-mail: Hassanvand@tums.ac.ir.

**Table S1**. Annual mean concentration of ambient air pollutants in Tehran during the study period (2012-2017).

| Year | $PM_{2.5}$ | $PM_{10}$ | $O_3$ | CO | $NO_2$ | $SO_2$ |
|---|---|---|---|---|---|---|
| **2012** | 36.02 | 86.27 | 20.67 | 2.46 | 35.58 | 20.45 |
| **2013** | 35.14 | 89.91 | 20.11 | 2.54 | 36.38 | 17.70 |
| **2014** | 32.58 | 78.81 | 19.42 | 2.67 | 44.54 | 19.50 |
| **2015** | 30.57 | 80.49 | 18.08 | 2.49 | 48.16 | 16.40 |
| **2016** | 30.25 | 88.64 | 17.48 | 2.65 | 52.53 | 11.91 |
| **2017** | 31.50 | 83.26 | 20.59 | 2.55 | 53.34 | 7.89 |

Units: µg $m^{-3}$ for $PM_{2.5}$, $PM_{10}$, ppb for $O_3$, $NO_2$ and $SO_2$, ppm for CO.

**Table S2.** Regression analysis model for seasonal variation of ambient air pollutant concentrations during the study period (2012-2017) in Tehran*.

| Air Pollutants | | Coef. | Std. Err. | t | P>\|t\| | [95% Conf. Interval] | |
|---|---|---|---|---|---|---|---|
| | Summer | 4.81115 | 0.18789 | 25.6 | 0.00 | 4.44286 | 5.17944 |
| **$PM_{2.5}$** | Fall | 6.93813 | 0.18941 | 36.63 | 0.00 | 6.56688 | 7.30938 |
| | Winter | 8.57391 | 0.19203 | 44.65 | 0.00 | 8.19752 | 8.95029 |
| | Summer | 19.77771 | 0.46262 | 42.75 | 0.00 | 18.87091 | 20.6844 |
| **$PM_{10}$** | Fall | 15.06674 | 0.46636 | 32.31 | 0.00 | 14.15265 | 15.9808 |
| | Winter | 10.02922 | 0.46716 | 21.47 | 0.00 | 9.11358 | 10.9448 |
| | Summer | 6.70620 | 0.18430 | 36.39 | 0.00 | 6.34496 | 7.06744 |
| **$NO_2$** | Fall | 7.38209 | 0. 18575 | 39.74 | 0.00 | 7.01800 | 7.74618 |
| | Winter | 8.70420 | 0. 18611 | 46.77 | 0.00 | 8.33942 | 9.06898 |
| | Summer | 2.09358 | 0.16066 | 13.03 | 0.00 | 1.77868 | 2.40849 |
| **$O_3$** | Fall | -9.13052 | 0.16235 | -56.24 | 0.00 | -9.44874 | -8.81230 |
| | Winter | -8.31545 | 0.16246 | -51.18 | 0.00 | -8.63388 | -7.99701 |
| | Summer | 0.48259 | 0.08571 | 5.63 | 0.00 | 0.31458 | 0.65060 |
| **$SO_2$** | Fall | 0.63586 | 0.08567 | 7.42 | 0.00 | 0.46794 | 0.80379 |
| | Winter | 4.42386 | 0.08587 | 51.51 | 0.00 | 4.25554 | 4.59219 |
| | Summer | 0.27357 | 0.01160 | 23.58 | 0.00 | 0.25082 | 0.29631 |
| **CO** | Fall | 0.56502 | 0.01169 | 48.31 | 0.00 | 0.54210 | 0.58795 |
| | Winter | 0.45855 | 0.01171 | 39.13 | 0.00 | 0.43558 | 0.48152 |

* The average concentration of each air pollutant in spring during the entire study period ($PM_{2.5}$: 27.63 (µg $m^{-3}$), $PM_{10}$ (µg $m^{-3}$): 73.37 ppb, $NO_2$: 39.43 ppb, $O_3$: 23.13 ppb, $SO_2$: 14.24 ppb, CO: 2.23 ppm) was considered as base category.

**Table S3.** Regression analysis model for monthly variation of $PM_{2.5}$ concentrations (µg $m^{-3}$) based on hourly data during the study period (2012-2017) in Tehran*.

| | Coef. | Std. Err. | t | P>\|t\| | [95% Conf. Interval] | |
|---|---|---|---|---|---|---|
| **January** | -6.355231 | 0.3199761 | -19.86 | 0.00 | -6.982387 | -5.728071 |
| **February** | -10.46956 | 0.3279314 | -31.93 | 0.00 | -11.11231 | -9.826812 |
| **March** | -17.40550 | 0.3200144 | -54.39 | 0.00 | -18.03273 | -16.77826 |
| **April** | -18.93596 | 0.3204378 | -59.09 | 0.00 | -19.56402 | -18.30790 |
| **May** | -13.61914 | 0.3164215 | -43.04 | 0.00 | -14.23933 | -12.99895 |
| **June** | -9.405079 | 0.3191790 | -29.47 | 0.00 | -10.03067 | -8.779485 |
| **July** | -9.291993 | 0.3164569 | -29.36 | 0.00 | -9.912252 | -8.671735 |
| **August** | -12.61476 | 0.3164746 | -39.86 | 0.00 | -13.23505 | -11.99447 |
| **September** | -12.14005 | 0.3191225 | -38.04 | 0.00 | -12.76553 | -11.51457 |
| **October** | -11.61322 | 0.3164392 | -36.70 | 0.00 | -12.23344 | -10.99299 |
| **November** | -8.669711 | 0.3190474 | -27.17 | 0.00 | -9.295047 | -8.044375 |

* The average concentration of $PM_{2.5}$ in December during the entire study period (43.48) was considered as base category.

**Table S4.** Regression analysis model for monthly variation of $PM_{10}$ concentrations (µg $m^{-3}$) based on hourly data during the study period (2012-2017) in Tehran*.

| | Coef. | Std. Err. | t | P>\|t\| | [95% Conf. Interval] | |
|---|---|---|---|---|---|---|
| **January** | -14.51451 | 0.786381 | -18.46 | 0.000 | -16.0558 | -12.9732 |
| **February** | -17.32962 | 0.804867 | -21.53 | 0.000 | -18.9071 | -15.7520 |
| **March** | -33.64300 | 0.786645 | -42.77 | 0.000 | -35.1848 | -32.1011 |
| **April** | -30.43641 | 0.792903 | -38.39 | 0.000 | -31.9905 | -28.8823 |
| **May** | -20.62778 | 0.786778 | -26.22 | 0.000 | -22.1698 | -19.0856 |
| **June** | -4.14537 | 0.793464 | -5.22 | 0.000 | -5.70057 | -2.59017 |
| **August** | -6.96299 | 0.787043 | -8.85 | 0.000 | -8.50560 | -5.42037 |
| **September** | -6.31216 | 0.793511 | -7.95 | 0.000 | -7.86745 | -4.75687 |
| **October** | -6.08185 | 0.786778 | -7.73 | 0.000 | -7.62394 | -4.53975 |
| **November** | -11.61251 | 0.793136 | -14.64 | 0.000 | -13.1670 | -10.0579 |
| **December** | -4.33473 | 0.786425 | -5.51 | 0.000 | -5.87613 | -2.79333 |

* The average concentration of $PM_{10}$ in July during the entire study period (94.59) was considered as base category.

**Table S5.** Regression analysis model for monthly variation of $NO_2$ concentrations (ppb) based on hourly data during the study period (2012-2017) in Tehran*.

| | Coef. | Std. Err. | t | P>\|t\| | [95% Conf. Interval] | |
|---|---|---|---|---|---|---|
| **January** | -1.277769 | 0.3171748 | -4.03 | 0.00 | -1.899435 | -0.656103 |
| **February** | -4.488716 | 0.3245520 | -13.83 | 0.00 | -5.124841 | -3.852592 |
| **March** | -8.978878 | 0.3171748 | -28.31 | 0.00 | -9.600544 | -8.357213 |
| **April** | -11.42728 | 0.3198070 | -35.73 | 0.00 | -12.05410 | -10.80045 |
| **May** | -12.27204 | 0.3171748 | -38.69 | 0.00 | -12.89370 | -11.65037 |
| **June** | -9.595833 | 0.3198070 | -30.01 | 0.00 | -10.22266 | -8.969008 |
| **July** | -6.573633 | 0.3171748 | -20.73 | 0.00 | -7.195299 | -5.951968 |
| **August** | -4.004784 | 0.3175673 | -12.61 | 0.00 | -4.627218 | -3.382349 |
| **September** | -2.840226 | 0.3198070 | -8.88 | 0.00 | -3.467051 | -2.213402 |
| **October** | -5.700551 | 0.3171748 | -17.97 | 0.00 | -6.322216 | -5.078885 |
| **November** | -4.062399 | 0.3198070 | -12.7 | 0.00 | -4.689224 | -3.435574 |

* The average concentration of $NO_2$ in December during the entire study period (52.02) was considered as base category.

**Table S6.** Regression analysis model for monthly variation of $O_3$ concentrations (ppb) based on hourly data during the study period (2012-2017) in Tehran*.

| | Coef. | Std. Err. | t | P>\|t\| | [95% Conf. Interval] | |
|---|---|---|---|---|---|---|
| **January** | -12.33160 | 0.2757187 | -44.73 | 0.000 | -12.8720 | -11.79119 |
| **February** | -11.69460 | 0.2826308 | -41.38 | 0.000 | -12.2485 | -11.14064 |
| **March** | -7.441589 | 0.2759518 | -26.97 | 0.000 | -7.98245 | -6.900721 |
| **April** | -5.821149 | 0.2779007 | -20.95 | 0.000 | -6.36583 | -5.276461 |
| **May** | -3.113485 | 0.2755949 | -11.3 | 0.000 | -3.65365 | -2.57331 |
| **July** | -0.236871 | 0.2778844 | -0.85 | 0.394 | -0.78152 | 0.307784 |
| **August** | -1.449024 | 0.2755486 | -5.26 | 0.000 | -1.98910 | -0.90894 |
| **September** | -5.326496 | 0.2778353 | -19.17 | 0.000 | -5.87105 | -4.781936 |
| **October** | -11.06699 | 0.2755486 | -40.16 | 0.000 | -1.16070 | -10.52691 |
| **November** | -13.97739 | 0.2779335 | -50.29 | 0.000 | -1.45221 | -13.43264 |
| **December** | -15.51331 | 0.2772351 | -55.96 | 0.000 | -16.0566 | -14.96993 |

* The average concentration of $O_3$ in June during the entire study period (26.69) was considered as base category.

**Table S7.** Regression analysis model for monthly variation of $SO_2$ concentrations (ppb) based on hourly data during the study period (2012-2017) in Tehran*.

| | Coef. | Std. Err. | t | P>\|t\| | [95% Conf. Interval] | |
|---|---|---|---|---|---|---|
| **February** | -1.862641 | 0.1495334 | -12.46 | 0.00 | -2.155728 | -1.569554 |
| **March** | -4.635948 | 0.1461345 | -31.72 | 0.00 | -4.922373 | -4.349523 |
| **April** | -6.399849 | 0.1473472 | -43.43 | 0.00 | -6.688651 | -6.111047 |
| **May** | -5.962407 | 0.1461918 | -40.78 | 0.00 | -6.248944 | -5.675869 |
| **June** | -6.486361 | 0.149116 | -43.50 | 0.00 | -6.778630 | -6.194092 |
| **July** | -6.142489 | 0.1471402 | -41.75 | 0.00 | -6.430885 | -5.854092 |
| **August** | -5.059057 | 0.1470888 | -34.39 | 0.00 | -5.347352 | -4.770761 |
| **September** | -4.803826 | 0.1476352 | -32.54 | 0.00 | -5.093193 | -4.514460 |
| **October** | -6.454409 | 0.1461345 | -44.17 | 0.00 | -6.740834 | -6.167984 |
| **November** | -5.534941 | 0.1473472 | -37.56 | 0.00 | -5.823743 | -5.246139 |
| **December** | -3.733180 | 0.1461345 | -25.55 | 0.00 | -4.019605 | -3.446755 |

* The average concentration of $SO_2$ in January during the entire study period (20.36) was considered as base category.

**Table S8.** Regression analysis model for monthly variation of CO concentrations (ppm) based on hourly data during the study period (2012-2017) in Tehran*.

| | Coef. | Std. Err. | t | P>\|t\| | [95% Conf. Interval] | |
|---|---|---|---|---|---|---|
| **January** | -0.0614042 | 0.0199977 | -3.07 | 0.002 | -0.100599 | -0.022208 |
| **February** | -0.1661982 | 0.0204624 | -8.12 | 0.00 | -0.206304 | -0.126091 |
| **March** | -0.5798126 | 0.0200168 | -28.97 | 0.00 | -0.619045 | -0.540579 |
| **April** | -0.6515720 | 0.0201635 | -32.31 | 0.00 | -0.691092 | -0.612051 |
| **May** | -0.6034431 | 0.0199977 | -30.18 | 0.00 | -0.642638 | -0.564247 |
| **June** | -0.5528730 | 0.0201635 | -27.42 | 0.00 | -0.592393 | -0.513352 |
| **July** | -0.3572207 | 0.0199977 | -17.86 | 0.00 | -0.396416 | -0.318025 |
| **August** | -0.3335618 | 0.0200315 | -16.65 | 0.00 | -0.372823 | -0.294299 |
| **September** | -0.2319705 | 0.0201635 | -11.5 | 0.00 | -0.271491 | -0.192449 |
| **October** | -0.0730040 | 0.0199977 | -3.65 | 0.00 | -0.112199 | -0.033808 |
| **November** | -0.0237707 | 0.0201635 | -1.18 | 0.238 | -0.063291 | 0.015750 |

* The average concentration of CO in December during the entire study period (2:86) was considered as base category.

**Table S9.** Regression analysis model for ambient air pollutant concentrations during the Nowruz holidays compared to the rest of year based on hourly data over the study period (2012-2017) in Tehran*.

| Air Pollutants | Coef. | Std. Err. | t | P>\|t\| | [95% Conf. Interval] | |
|---|---|---|---|---|---|---|
| **$PM_{2.5}$** | -15.88912 | 0.34940 | -45.47 | 0.000 | -16.57396 | -15.20427 |
| **$PM_{10}$** | -45.27450 | 0.85266 | -53.10 | 0.000 | -46.94573 | -43.60327 |
| **$NO_2$** | -8.41359 | 0. 34896 | -24.11 | 0.000 | -9.097565 | -7.729632 |
| **$O_3$** | 3.43363 | 0. 32045 | 10.71 | 0.000 | 2.805535 | 4.061728 |
| **$SO_2$** | -1.11102 | 0. 16276 | -6.83 | 0.000 | -1.430040 | -0.792001 |
| **CO** | -0.44940 | 0. 02201 | -20.42 | 0.000 | -0.492548 | -0.406267 |

* The average concentration of each air pollutant in rest of year ($PM_{2.5}$: 33.3 (µg $m^{-3}$), $PM_{10:}$ 88.3 (µg $m^{-3}$), $NO_2$: 45.4 ppb, $O_3$: 19.3 ppb, $SO_2$: 15.7 ppb, CO: 2.6 ppm) was considered as base category.

**Table S10.** Regression analysis model for mean $PM_{2.5}$ concentrations ($\mu g\ m^{-3}$) on weekends and weekdays based on hourly data during the study period (2012-2017) in Tehran*.

| Weekdays | Coef. | Std. Err. | t | P>\|t\| | [95% Conf. Interval] | |
|---|---|---|---|---|---|---|
| **Saturday** | 1.477507 | 0.2568953 | 5.75 | 0.000 | 0.9739902 | 1.981025 |
| **Sunday** | 2.624146 | 0.2567139 | 10.22 | 0.000 | 2.120985 | 3.127308 |
| **Monday** | 3.166912 | 0.2569994 | 12.32 | 0.000 | 2.663191 | 3.670633 |
| **Tuesday** | 3.339201 | 0.2566881 | 13.01 | 0.000 | 2.83609 | 3.842313 |
| **Wednesday** | 3.561203 | 0.2567139 | 13.87 | 0.000 | 3.058042 | 4.064365 |
| **Thursday** | 2.224629 | 0.2571124 | 8.65 | 0.000 | 1.720686 | 2.728571 |

* The average concentration of $PM_{2.5}$ on Fridays during the entire study period (30.29) was considered as base category.

**Table S11.** Regression analysis model for mean $PM_{10}$ concentrations ($\mu g\ m^{-3}$) on weekends and weekdays based on hourly data during the study period (2012-2017) in Tehran*.

| Weekdays | Coef. | Std. Err. | t | P>\|t\| | [95% Conf. Interval] | |
|---|---|---|---|---|---|---|
| **Saturday** | 5.700221 | 0.6264094 | 9.10 | 0.000 | 4.472.452 | 6.927989 |
| **Sunday** | 8.338171 | 0.6261391 | 13.32 | 0.000 | 7.110.933 | 9.565410 |
| **Monday** | 9.253535 | 0.6268896 | 14.76 | 0.000 | 8.024.826 | 10.48224 |
| **Tuesday** | 10.80456 | 0.6264927 | 17.25 | 0.000 | 9.576627 | 12.03249 |
| **Wednesday** | 11.74322 | 0.6266387 | 18.74 | 0.000 | 10.51500 | 12.97144 |
| **Thursday** | 8.329395 | 0.6266596 | 13.29 | 0.000 | 7.101137 | 9.557654 |

* The average concentration of $PM_{10}$ on Fridays during the entire study period (76.83) was considered as base category.

**Table S12**. Regression analysis model for hourly variation of $PM_{2.5}$ concentrations ($\mu g\ m^{-3}$) during the study period (2012-2017) in Tehran*.

| | Coef. | Std. Err. | t | P>t | [95% Conf. Interval] | |
|---|---|---|---|---|---|---|
| **1** | -0.292649 | 0.470101 | -0.62 | 0.530 | -1.214053 | 0.628755 |
| **2** | -0.764550 | 0.470047 | -1.63 | 0.100 | -1.685848 | 0.156747 |
| **3** | -1.699044 | 0.470047 | -3.61 | 0.000 | -2.620342 | -0.777746 |
| **4** | -2.630836 | 0.470048 | -5.60 | 0.000 | -3.552134 | -1.709538 |
| **5** | -3.175771 | 0.470048 | -6.76 | 0.000 | -4.097069 | -2.254473 |
| **6** | -2.966130 | 0.470047 | -6.31 | 0.000 | -3.887428 | -2.044832 |
| **7** | -1.856729 | 0.470047 | -3.95 | 0.000 | -2.778027 | -0.935431 |
| **8** | -1.129328 | 0.470047 | -2.40 | 0.020 | -2.050625 | -0.208029 |
| **9** | -1.607595 | 0.470047 | -3.42 | 0.000 | -2.528893 | -0.686297 |
| **10** | -2.668769 | 0.470047 | -5.68 | 0.000 | -3.590067 | -1.747471 |
| **11** | -3.822662 | 0.470047 | -8.13 | 0.000 | -4.743959 | -2.90136 |
| **12** | -5.134876 | 0.470048 | -10.92 | 0.000 | -6.056174 | -4.213579 |
| **13** | -6.288844 | 0.470046 | -13.38 | 0.000 | -7.210142 | -5.367546 |
| **14** | -7.249698 | 0.470048 | -15.42 | 0.000 | -8.170996 | -6.328411 |
| **15** | -7.880386 | 0.470156 | -16.76 | 0.000 | -8.801897 | -6.958875 |
| **16** | -8.074576 | 0.470156 | -17.17 | 0.000 | -8.996087 | -7.153066 |
| **17** | -7.991190 | 0.470048 | -17.00 | 0.000 | -8.912488 | -7.069892 |
| **18** | -7.406609 | 0.470157 | -15.75 | 0.000 | -8.328120 | -6.485098 |
| **19** | -6.051507 | 0.470047 | -12.87 | 0.000 | -6.972805 | -5.130209 |
| **20** | -4.272127 | 0.470102 | -9.09 | 0.000 | -5.193531 | -3.350722 |
| **21** | -2.655527 | 0.470211 | -5.65 | 0.000 | -3.577145 | -1.733917 |
| **22** | -1.097550 | 0.470265 | -2.33 | 0.020 | -2.019273 | -0.175826 |
| **23** | -0.481747 | 0.453269 | -1.06 | 0.290 | -1.370101 | 0.406692 |

* The average concentration of $PM_{2.5}$ at 00:00 o'clock during the entire study period (36.27) was considered as base category.

**Table S13**. Regression analysis model for hourly variation of $PM_{10}$ concentrations ($\mu g\ m^{-3}$) during the study period (2012-2017) in Tehran*.

| | Coef. | Std. Err. | t | P>t | [95% Conf. Interval] | |
|---|---|---|---|---|---|---|
| **1** | -1.696534 | 1.153396 | -1.47 | 0.140 | -3.957202 | 0.564133 |
| **2** | -3.950163 | 1.153133 | -3.43 | 0.000 | -6.210314 | -1.690012 |
| **3** | -7.061503 | 1.152871 | -6.13 | 0.000 | -9.321139 | -4.801868 |
| **4** | -10.27187 | 1.152607 | -8.91 | 0.000 | -12.53099 | -8.012745 |
| **5** | -12.43362 | 1.152607 | -10.79 | 0.000 | -14.69275 | -10.174512 |
| **6** | -11.42965 | 1.152607 | -9.92 | 0.000 | -13.68877 | -9.170529 |
| **7** | -7.529318 | 1.152607 | -6.53 | 0.000 | -9.788438 | -5.270197 |
| **8** | -6.809341 | 1.152607 | -5.91 | 0.000 | -9.068461 | -4.550224 |
| **9** | -8.408498 | 1.152872 | -7.29 | 0.000 | -10.66813 | -6.148862 |
| **10** | -9.364423 | 1.152871 | -8.12 | 0.000 | -11.62406 | -7.104788 |
| **11** | -10.59223 | 1.152738 | -9.19 | 0.000 | -12.85161 | -8.332854 |
| **12** | -13.07172 | 1.152872 | -11.34 | 0.000 | -15.33135 | -10.812081 |
| **13** | -15.87413 | 1.152872 | -13.77 | 0.000 | -18.13377 | -13.614511 |
| **14** | -18.12904 | 1.152872 | -15.73 | 0.000 | -20.38867 | -15.869411 |
| **15** | -18.96473 | 1.152872 | -16.45 | 0.000 | -21.22436 | -16.705091 |
| **16** | -18.32226 | 1.152872 | -15.89 | 0.000 | -20.58189 | -16.062621 |
| **17** | -17.09542 | 1.152872 | -14.83 | 0.000 | -19.35506 | -14.835791 |
| **18** | -15.44339 | 1.153001 | -13.39 | 0.000 | -177.0329 | -13.183577 |
| **19** | -12.48697 | 1.153265 | -10.83 | 0.000 | -14.747317 | -10.226491 |
| **20** | -8.244695 | 1.153528 | -7.15 | 0.000 | -10.505677 | -5.983769 |
| **21** | -5.074532 | 1.153925 | -4.40 | 0.000 | -7.336235 | -2.812829 |
| **22** | -2.267403 | 1.153925 | -1.96 | 0.050 | -4.529106 | -0.005700 |
| **23** | -0.258772 | 1.153661 | -0.22 | 0.820 | -2.519958 | 2.002412 |

* The average concentration of $PM_{10}$ at 00:00 o'clock during the entire study period (94.36) was considered as base category.

**Table S14.** Regression analysis model for mean concentrations of air pollutants during daytime (8:00-21:00) in comparison with their concentrations at night time (22:00-7:00) based on hourly data during the study period (2012-2017) in Tehran*.

| Air Pollutants | Coef. | Std. Err. | t | P>\|t\| | [95% Conf. Interval] | |
|---|---|---|---|---|---|---|
| **$PM_{2.5}$** | -3.67277 | 0.138662 | -26.49 | 0.000 | -3.944554 | -3.400993 |
| **$PM_{10}$** | -7.01146 | 0.33969 | -20.64 | 0.000 | -7.677263 | -6.345669 |
| **$NO_2$** | -3.26611 | 0.13591 | -24.03 | 0.000 | -3.532510 | -2.999723 |
| **$O_3$** | 1.27595 | 0.11123 | 114.71 | 0.000 | 1.2541510 | 1.297755 |
| **$SO_2$** | 1.02356 | 0.06352 | 16.11 | 0.000 | 0.8990465 | 1.148082 |
| **CO** | -0.53339 | 0.00828 | -64.36 | 0.000 | -0.549637 | -0.517147 |

* The average night time concentration of air pollutants during the entire study period (22:00-7:00) ($PM_{2.5}$: 34.8 ($\mu g\ m^{-3}$), $PM_{10}$: 88.7 ($\mu g\ m^{-3}$), $NO_2$: 47.0 (ppb), $O_3$: 11.9 (ppb), $SO_2$: 24.1 (ppb), CO: 2.9 (ppm)) was considered as base category.

**Table S15.** Regression analysis model for mean $NO_2$ concentrations (ppb) on weekends and weekdays based on hourly data during the study period (2012-2017) in Tehran*.

| Week Days | Coef. | Std. Err. | t | P>\|t\| | [95% Conf. Interval] | |
|---|---|---|---|---|---|---|
| **Saturday** | 3.197351 | 0.2514286 | 12.72 | 0.000 | 2.704549 | 3.690153 |
| **Sunday** | 3.169046 | 0.2510441 | 12.62 | 0.000 | 26.76998 | 3.661095 |
| **Monday** | 3.701830 | 0.2512442 | 14.73 | 0.000 | 3.209389 | 4.194271 |
| **Tuesday** | 3.859317 | 0.2512442 | 15.36 | 0.000 | 3.366876 | 4.351758 |
| **Wednesday** | 3.914307 | 0.2512442 | 15.58 | 0.000 | 3.421866 | 4.406748 |
| **Thursday** | 3.500397 | 0.2512442 | 13.93 | 0.000 | 3.007956 | 3.992838 |

* The average concentration of $NO_2$ on Fridays during the entire study period (42.03) was considered as base category.

**Table S16.** Regression analysis model for mean $O_3$ concentrations (ppb) on weekends and weekdays based on hourly data during the study period (2012-2017) in Tehran*.

| Week Days | Coef. | Std. Err. | t | P>\|t\| | [95% Conf. Interval] | |
|---|---|---|---|---|---|---|
| **Saturday** | -1.578988 | 0.2290194 | -6.89 | 0.000 | -2.02786 | -1.13010 |
| **Sunday** | -1.737033 | 0.2288290 | -7.59 | 0.000 | -2.18554 | -1.288527 |
| **Monday** | -1.762067 | 0.2291726 | -7.69 | 0.000 | -2.21124 | -1.312887 |
| **Tuesday** | -1.703171 | 0.2291573 | -7.43 | 0.000 | -2.15232 | -1.254021 |
| **Wednesday** | -1.560491 | 0.2293574 | -6.80 | 0.000 | -2.01003 | -1.110949 |
| **Thursday** | -1.421886 | 0.2292957 | -6.20 | 0.000 | -1.87130 | -0.972464 |

* The average concentration of $O_3$ on Fridays during the entire study period (20.79) was considered as base category.

**Table S17.** Regression analysis model for mean $SO_2$ concentrations (ppb) on weekends and weekdays based on hourly data during the study period (2012-2017) in Tehran*.

| Week Days | Coef. | Std. Err. | t | P>\|t\| | [95% Conf. Interval] | |
|---|---|---|---|---|---|---|
| **Saturday** | 0.5609136 | 0.1174015 | 4.78 | 0.000 | 0.3308055 | 0.7910217 |
| **Sunday** | 0.6030873 | 0.1175127 | 5.13 | 0.000 | 0.3727612 | 0.8334134 |
| **Monday** | 0.8642319 | 0.1174174 | 7.36 | 0.000 | 0.6340927 | 1.0943710 |
| **Tuesday** | 1.1483230 | 0.1171189 | 9.80 | 0.000 | 0.9187691 | 1.3778780 |
| **Wednesday** | 1.1703110 | 0.1172048 | 9.99 | 0.000 | 0.9405883 | 1.4000330 |
| **Thursday** | 0.7396296 | 0.1172322 | 6.31 | 0.000 | 0.5098534 | 0.9694058 |

* The average concentration of $SO_2$ on Fridays during the entire study period (14.88) was considered as base category.

**Table S18.** Regression analysis model for mean CO concentrations (ppm) on weekends and weekdays based on hourly data during the study period (2012-2017) in Tehran*.

| Week Days | Coef. | Std. Err. | t | P>\|t\| | [95% Conf. Interval] | |
|---|---|---|---|---|---|---|
| **Saturday** | 0.1481885 | 0.015860 | 9.34 | 0.000 | 0.1171027 | 0.1792742 |
| **Sunday** | 0.1290408 | 0.015848 | 8.14 | 0.000 | 0.0979777 | 0.1601038 |
| **Monday** | 0.1608120 | 0.015861 | 10.14 | 0.000 | 0.1297231 | 0.1919009 |
| **Tuesday** | 0.1653927 | 0.015858 | 10.43 | 0.000 | 0.1343100 | 0.1964754 |
| **Wednesday** | 0.1749174 | 0.015861 | 11.03 | 0.000 | 0.1438285 | 0.2060063 |
| **Thursday** | 0.1412876 | 0.015859 | 8.91 | 0.000 | 0.1102039 | 0.1723713 |

* The average concentration of CO on Fridays during the entire study period (2.43) was considered as base category.

**Table S19**. Regression analysis model for hourly variation of $NO_2$ concentrations (ppb) during the study period (2012-2017) in Tehran*.

| | **Coef.** | **Std. Err.** | **t** | **P>t** | **[95% Conf. Interval]** | |
|---|---|---|---|---|---|---|
| **0** | -2.95668 | 0.4428 | -6.68 | 0.000 | -3.824585 | -2.088787 |
| **1** | -4.85706 | 0.44280 | -10.97 | 0.000 | -5.724966 | -3.989167 |
| **2** | -6.63818 | 0.44285 | -14.99 | 0.000 | -7.506182 | -5.770185 |
| **3** | -8.35665 | 0.44285 | -18.87 | 0.000 | -9.224654 | -7.488657 |
| **4** | -9.10248 | 0.44285 | -20.55 | 0.000 | -9.970485 | -8.234488 |
| **5** | -8.53143 | 0.44285 | -19.26 | 0.000 | -9.399434 | -7.663437 |
| **6** | -6.42998 | 0.44285 | -14.52 | 0.000 | -7.297983 | -5.561986 |
| **7** | -3.84372 | 0.44285 | -8.68 | 0.000 | -4.711723 | -2.975726 |
| **8** | -2.63474 | 0.44285 | -5.95 | 0.000 | -3.502746 | -1.766749 |
| **9** | -3.62742 | 0.44285 | -8.19 | 0.000 | -4.495423 | -2.759426 |
| **10** | -6.47907 | 0.44285 | -14.63 | 0.000 | -7.347071 | -5.611074 |
| **11** | -9.57704 | 0.44285 | -21.63 | 0.000 | -1.044504 | -8.709044 |
| **12** | -12.55828 | 0.44285 | -28.36 | 0.000 | -1.342627 | -1.169028 |
| **13** | -14.80479 | 0.44285 | -33.43 | 0.000 | -1.567279 | -1.393679 |
| **14** | -15.97445 | 0.44285 | -36.07 | 0.000 | -1.684245 | -1.510645 |
| **15** | -15.86715 | 0.44285 | -35.83 | 0.000 | -1.673515 | -1.499915 |
| **16** | -14.44373 | 0.44285 | -32.62 | 0.000 | -1.531173 | -1.357574 |
| **17** | -11.58362 | 0.44285 | -26.16 | 0.000 | -1.245162 | -1.071562 |
| **18** | -7.759577 | 0.44285 | -17.52 | 0.000 | -8.627576 | -6.891579 |
| **19** | -3.70657 | 0.44285 | -8.37 | 0.000 | -4.574568 | -2.838572 |
| **20** | -0.71475 | 0.44285 | -1.61 | 0.110 | -1.582750 | 0.153247 |
| **22** | -0.62090 | 0.44285 | -1.40 | 0.160 | -1.488912 | 0.247097 |
| **23** | -1.52530 | 0.44285 | -3.44 | 0.000 | -2.393306 | -0.657309 |

* The average concentration of $NO_2$ at 21:00 o'clock during the entire study period (52.28) was considered as base category.

**Table S20**. Regression analysis model for hourly variation of CO concentrations (ppm) during the study period (2012-2017) in Tehran*.

| | **Coef.** | **Std. Err.** | **t** | **P>t** | **[95% Conf. Interval]** | |
|---|---|---|---|---|---|---|
| **0** | -0.22590 | 0.0251 | -8.99 | 0.000 | -0.275157 | -0.176651 |
| **1** | -0.45620 | 0.0251 | -18.14 | 0.000 | -0.505490 | -0.406927 |
| **2** | -0.70456 | 0.0251 | -28.03 | 0.000 | -0.753829 | -0.655300 |
| **3** | -0.91937 | 0.0251 | -36.57 | 0.000 | -0.968640 | -0.870100 |
| **4** | -1.03739 | 0.0251 | -41.27 | 0.000 | -1.086666 | -0.988126 |
| **5** | -0.97233 | 0.0251 | -38.68 | 0.000 | -1.021607 | -0.923055 |
| **6** | -0.67325 | 0.0251 | -26.79 | 0.000 | -0.722518 | -0.624000 |
| **7** | -0.37279 | 0.0251 | -14.84 | 0.000 | -0.422043 | -0.323537 |
| **8** | -0.44038 | 0.0251 | -17.53 | 0.000 | -0.489640 | -0.391134 |
| **9** | -0.75943 | 0.0251 | -30.22 | 0.000 | -0.808687 | -0.710181 |
| **10** | -1.05933 | 0.0251 | -42.15 | 0.000 | -1.108611 | -1.010071 |
| **11** | -1.26795 | 0.0251 | -50.43 | 0.000 | -1.317234 | -1.218683 |
| **12** | -1.41557 | 0.0251 | -56.30 | 0.000 | -1.464851 | -1.366289 |
| **13** | -1.51591 | 0.0252 | -60.27 | 0.000 | -1.565208 | -1.466612 |
| **14** | -1.56902 | 0.0252 | -62.38 | 0.000 | -1.618322 | -1.519726 |
| **15** | -1.59411 | 0.0251 | -63.39 | 0.000 | -1.643412 | -1.544827 |
| **16** | -1.55509 | 0.0251 | -61.86 | 0.000 | -1.604363 | -1.505823 |
| **17** | -1.41940 | 0.0251 | -56.48 | 0.000 | -1.468660 | -1.370142 |
| **18** | -1.15219 | 0.0251 | -45.85 | 0.000 | -1.201447 | -1.102941 |
| **19** | -0.77223 | 0.0251 | -30.73 | 0.000 | -0.821487 | -0.722980 |
| **20** | -0.39893 | 0.0251 | -15.88 | 0.000 | -0.448189 | -0.349683 |
| **21** | -0.12706 | 0.0251 | -5.06 | 0.000 | -0.176322 | -0.077816 |
| **23** | -0.04886 | 0.0251 | -1.94 | 0.050 | -0.098117 | 0.000389 |

* The average concentration of CO at 22:00 o'clock during the entire study period (3.41) was considered as base category.

**Table S21**. Regression analysis model for hourly variation of $O_3$ concentrations (ppb) during the study period (2012-2017) in Tehran*.

| | **Coef.** | **Std. Err.** | **t** | **P>t** | **[95% Conf. Interval]** | |
|---|---|---|---|---|---|---|
| **0** | -23.93368 | 0.3337126 | -71.72 | 0.000 | -24.58776 1 | -23.279612 |
| **1** | -23.66222 | 0.3337509 | -70.90 | 0.000 | -24.316371 | -23.008016 |
| **2** | -23.17303 | 0.3339431 | -69.39 | 0.000 | -23.82756 1 | -22.518523 |
| **3** | -22.46389 | 0.3338277 | -67.29 | 0.000 | -23.118211 | -21.809591 |
| **4** | -22.00031 | 0.3338662 | -65.90 | 0.000 | -22.654691 | -21.345931 |
| **5** | -21.69152 | 0.3335213 | -65.04 | 0.000 | -22.34522 3 | -21.037811 |
| **6** | -21.43816 | 0.3334832 | -64.29 | 0.000 | -22.091792 | -20.784532 |
| **7** | -20.36046 | 0.3334832 | -61.05 | 0.000 | -21.014094 | -19.706832 |
| **8** | -17.63445 | 0.3335213 | -52.87 | 0.000 | -18.288154 | -16.980752 |
| **9** | -13.59003 | 0. 3334832 | -40.75 | 0.000 | -14.243664 | -12.936423 |
| **10** | -9.271962 | 0. 3334832 | -27.80 | 0.000 | -9.925592 | -14.975721 |
| **11** | -5.423432 | 0. 3334832 | -16.26 | 0.000 | -6.077062 | -4.769802 |
| **12** | -2.348465 | 0. 3334450 | -14.12 | 0.000 | -3.002021 | -1.694911 |
| **13** | -0.481667 | 0. 3334451 | -1.44 | 0.149 | -1.135223 | 0.171887 |
| **15** | -1.127488 | 0. 3334450 | -3.38 | 0.001 | -1.781043 | -0.473932 |
| **16** | -3.884964 | 0. 3335213 | -11.65 | 0.000 | -4.538668 | -3.231259 |
| **17** | -8.187281 | 0. 3335595 | -24.55 | 0.000 | -8.841061 | -7.533501 |
| **18** | -13.23943 | 0. 3335595 | -39.69 | 0.000 | -13.893212 | -12.585658 |
| **19** | -18.15356 | 0. 3335595 | -54.42 | 0.000 | -18.807341 | -17.499781 |
| **20** | -21.58928 | 0. 3334832 | -64.74 | 0.000 | -22.242912 | -20.935658 |
| **21** | -23.34104 | 0. 3335213 | -69.98 | 0.000 | -23.99474 1 | -22.687338 |
| **22** | -23.74411 | 0. 3335213 | -71.19 | 0.000 | -24.39782 1 | -23.090418 |
| **23** | -23.87872 | 0. 3335595 | -71.59 | 0.000 | -29.697212 | -28.110988 |

* The average concentration of $O_3$ at 14:00 o'clock during the entire study period (34.10) was considered as base category.

**Table S22**. Regression analysis model for hourly variation of $SO_2$ concentrations (ppb) during the study period (2012-2017) in Tehran*.

| | **Coef.** | **Std. Err.** | **t** | **P>t** | **[95% Conf. Interval]** | |
|---|---|---|---|---|---|---|
| **0** | -1.80100 | 0.2159 | -8.34 | 0.000 | -2.224234 | -1.377774 |
| **1** | -2.07442 | 0.2159 | -9.61 | 0.000 | -2.497601 | -1.651239 |
| **2** | -2.51932 | 0.2160 | -11.66 | 0.000 | -2.942753 | -2.095903 |
| **3** | -2.80621 | 0.2161 | -12.98 | 0.000 | -3.229834 | -2.382594 |
| **4** | -3.06342 | 0.2161 | -14.18 | 0.000 | -3.486949 | -2.639904 |
| **5** | -3.00936 | 0.2161 | -13.93 | 0.000 | -3.432890 | -2.585846 |
| **6** | -2.49891 | 0.2160 | -11.57 | 0.000 | -2.922339 | -2.075490 |
| **7** | -1.88181 | 0.2160 | -8.71 | 0.000 | -2.305094 | -1.458537 |
| **8** | -1.34673 | 0.2158 | -6.24 | 0.000 | -1.769768 | -0.923696 |
| **9** | -0.78207 | 0.2159 | -3.62 | 0.000 | -1.205261 | -0.358898 |
| **10** | -0.09339 | 0.2158 | -0.43 | 0.670 | -0.516429 | 0.329649 |
| **11** | 0.18230 | 0.2159 | 0.84 | 0.400 | -0.240927 | 0.605532 |
| **13** | -0.60050 | 0.2159 | -2.78 | 0.010 | -1.023730 | -0.177270 |
| **14** | -1.16198 | 0.2159 | -5.38 | 0.000 | -1.585219 | -0.738759 |
| **15** | -1.60367 | 0.2159 | -7.43 | 0.000 | -2.026763 | -1.180595 |
| **16** | -1.97544 | 0.2159 | -9.15 | 0.000 | -2.398576 | -1.552311 |
| **17** | -2.13590 | 0.2160 | -9.89 | 0.000 | -2.559279 | -1.712527 |
| **18** | -2.17729 | 0.2161 | -10.08 | 0.000 | -2.600773 | -1.753826 |
| **19** | -2.14073 | 0.2160 | -9.91 | 0.000 | -2.564119 | -1.717358 |
| **20** | -1.88821 | 0.2160 | -8.74 | 0.000 | -2.311537 | -1.464883 |
| **21** | -1.62158 | 0.2160 | -7.51 | 0.000 | -2.044958 | -1.198206 |
| **22** | -1.47741 | 0.2160 | -6.84 | 0.000 | -1.900789 | -1.054037 |
| **23** | -1.49569 | 0.2159 | -6.93 | 0.000 | -1.918974 | -1.072418 |

* The average concentration of $SO_2$ at 12:00 o'clock during the entire study period (17.28) was considered as base category.

**Table S23.** The range of Air Quality Index (AQI) values in Tehran during the study period from 2012 to 2017.

| Years | Min | Max |
|---|---|---|
| **2012** | 71 | 489 |
| **2013** | 67 | 426 |
| **2014** | 65 | 497 |
| **2015** | 70 | 486 |
| **2016** | 63 | 451 |
| **2017** | 71 | 219 |

**Table S24**. Annual evolution of AQI for six criteria air pollutants based on U.S. EPA method during the study period (2012-2017).

| AQI subcategories | Number of days | | | | | |
|---|---|---|---|---|---|---|
| | **2012** | **2013** | **2014** | **2015** | **2016** | **2017** |
| **Good** | 0 | 0 | 0 | 0 | 0 | 0 |
| **Moderate** | 25 | 45 | 58 | 57 | 62 | 18 |
| **UFSG** | 189 | 185 | 164 | 185 | 207 | 238 |
| **Unhealthy** | 140 | 134 | 133 | 116 | 94 | 108 |
| **Very unhealthy** | 11 | 1 | 9 | 4 | 2 | 1 |
| **Hazardous** | 1 | 0 | 1 | 3 | 1 | 0 |

**Table S25.** Mann–Kendall trend test and Sen's slope estimator for inter-annual AQI subcategories during the study period (2012-2017), * P<0.05.

| Air pollutants | Mann-kendall trend (z ,s) | Sen's slope estimate (% change/year) |
|---|---|---|
| **Moderate** | 8* | 7.6 |
| **UFSG** | 6 | 9.8 |
| **Unhealthy** | -10* | -10.3 |
| **Very unhealthy** | -8 | -2.0 |
| **Hazardous** | -1 | 0.0 |

**Table S26.** Spearman's correlation coefficients (r) between air pollutants and meteorological parameters in Tehran (2012-2017) * P < 0.05.

| | $PM_{2.5}$ | $PM_{10}$ | $NO_2$ | CO | $SO_2$ | $O_3$ | Tem | RH | Pre | WS | Vis | Neb | SR |
|---|---|---|---|---|---|---|---|---|---|---|---|---|---|
| **Temperature (Tem)** | -0.124* | 0.323* | -0.172* | -0.296* | 0.126* | 0.626* | | | | | | | |
| **Relative humidity (RH)** | 0.128* | 0.356* | 0.121* | 0.322* | -0.149* | -0.677* | -0.796* | | | | | | |
| **Precipitation (Pre)** | -0.259 * | -0.392* | -0.007 | -0.113* | -0.191* | -0.346* | -0.345* | 0.597* | | | | | |
| **Wind speed (WS)** | -0.303* | -0.186* | -0.284* | -0.461* | -0.078 | 0.449* | 0.256* | -0.416* | -0.092* | | | | |
| **Visibility (Vis)** | -0.441* | -0.249* | -0.318* | -0.387* | -0.002 | 0.525* | 0.405* | -0.569* | -0.242* | 0.518* | | | |
| **Nebulosity (Neb)** | -0.016* | -0.136* | 0.190* | 0.197* | -0.273* | -0.441* | -0.400* | 0.511* | 0.384* | -0.356* | -0.434* | | |
| **Solar radiation (SR)** | -0.035* | 0.050 | -0.082 | -0.291* | -0.234* | 0.550* | 0.443* | -0.580* | -0.309* | 0.378* | 0.326* | -0.262* | |

**Table S27.** The information regarding Tehran Air Quality Monitoring Stations and hourly data coverage for air pollutants.

| No. | Station | Location | | | Hourly data coverage (%)* | | | | | |
|---|---|---|---|---|---|---|---|---|---|---|
| | Name | City district | Latitude | Longitude | $PM_{2.5}$ | $PM_{10}$ | $NO_2$ | $O_3$ | $SO_2$ | CO |
| 1 | Roz Park | 22 | 39.92 | 53.92 | 41.0-94.8 | 53.6-96.8 | 23.6-75.7 | 21.2-77.4 | 21.6-85.4 | 33.0-96.4 |
| 2 | Poonak | 5 | 35.76 | 51.33 | 70.2-83.6 | 47.1-81.7 | 54.9-86.4 | 14.2-74.9 | 44.0-75.2 | 51.9-84.0 |
| 3 | Zone 2 | 2 | 35.77 | 51.36 | 13.2-91.1 | 23.3-80.2 | 17.6-66.6 | 14.4-72.6 | 17.8-33.3 | 34.0-55.3 |
| 4 | Darous | 3 | 35.77 | 51.45 | 50.7-68.4 | 0.0-4.1 | 46.1-61.6 | 24.1-40.1 | 11.0-55.1 | 34.0-96.1 |
| 5 | Golbarg | 8 | 35.73 | 51.50 | 21.1- 91.2 | 65.0-92.2 | 18.3-91.4 | 30.0-88.2 | 23.9-83.9 | 50.1-97.4 |
| 6 | Setad bohran | 7 | 35.72 | 51.43 | 52.3-97.1 | 37.5-90.7 | 49.2-86.2 | 43.2-87.2 | 15.6-83.0 | 60.2-90.0 |
| 7 | Sharif | 2 | 35.70 | 51.35 | 55.8-97.0 | 57.0-97.7 | 56.7-94.3 | 32.2-99.8 | 14.4-65.1 | 47.6-67.0 |
| 8 | Tarbiat Modares | 6 | 35.71 | 51.38 | 49.6-96.6 | 54.0-97.1 | 74.4-99.0 | 48.3-78.2 | 26.5-98.5 | 30.2-97.6 |
| 9 | Piroozi | 13 | 35.69 | 51.49 | 19.2-88.5 | 20.9-97.0 | 47.5-96.9 | 20.0-84.3 | 14.7-97.7 | 11.3-97.8 |
| 10 | Fath | 9 | 35.67 | 51.33 | - | 64.2-92.3 | 47.5-89.4 | 29.7-87.9 | 15.4-56.2 | 21.6-65.2 |
| 11 | Zone 11 | 11 | 35.67 | 51.38 | 26.7-96.8 | 32.0-54.2 | 27.5-78.0 | 13.6-62.2 | 29.2-90.4 | 24.5-95.0 |
| 12 | Zone 16 | 16 | 35.64 | 51.39 | 29.8-73.9 | 64.0-8٠.1 | 51.4-95.7 | 36.3-82.3 | 44.5-81.4 | 54.2-96.8 |
| 13 | Shad abad | 18 | 35.67 | 51.29 | 79.7-99.2 | 64.6-97.6 | 35.0-97.7 | 50.4-97.1 | 22.4-91.2 | 30.8-69.0 |
| 14 | Zone 19 | 19 | 35.63 | 51.36 | 31.0-61.1 | 34.3-80.2 | 28.5-73.0 | 17.3-49.2 | 28.1-53.4 | 29.0-81.1 |
| 15 | Masoudiyeh | 15 | 35.63 | 51.49 | 18.3-93.9 | 11.9-91.9 | 32.9-69.5 | 20.9-73.5 | 11.4-88.9 | 30.1-97.7 |
| 16 | Share rey | 20 | 35.60 | 51.42 | 13.5-96.9 | 45.0-88.7 | 34.5-81.1 | 41.2-88.4 | 32.3-91.1 | 73.0-97.1 |
| 17 | Zone 4 | 4 | 35.74 | 51.50 | 35.9-75.1 | 46.1-78.6 | 46.2-88.7 | 40.3-76.2 | 23.1-80.0 | 59.7-91.3 |
| 18 | Zone 10 | 10 | 35.69 | 51.35 | 35.2-91.0 | 41.1-55.0 | 45.1-49.0 | 39.1-67.0 | 55.6-93.5 | 58.4-73.0 |
| 19 | Mahalati | 14 | 35.66 | 51.46 | - | 46.3-93.7 | 39.8-69.2 | 13.1-68.8 | 13.8-51.8 | 11.9-49.5 |
| 20 | Tehransar | 21 | 35.69 | 51.24 | 20.1-90.6 | 23.1-92.2 | 21.2-92.8 | 19.0-69.1 | 22.3-92.4 | 11.5-40.8 |
| 21 | Aghdasiyeh | 1 | 35.79 | 51.48 | 55.8-92.6 | 29.1-93.9 | 45.8-82.0 | 27.6-69.1 | 19.6-62.2 | 59.4-98.3 |

* Air quality monitoring stations with less than 70% valid hourly data available in each year were excluded from our analysis.

**Table S28.** Statistical summary of meteorological data in Tehran during the study period (2012-2017).

| MD [a] | Descriptive | 2012 | 2013 | 2014 | 2015 | 2016 | 2017 |
|---|---|---|---|---|---|---|---|
| **Tem [b] (°C)** | Min [h] | -2.7 | -1.1 | -6.4 | 0.7 | -5.2 | -1.0 |
| | Max [i] | 32.3 | 36.0 | 35.0 | 35.1 | 34.0 | 33.7 |
| | Ave [j] ± SD [k] | 16.7±10.1 | 17.8±9.6 | 17.5±10.3 | 17.9±9.9 | 17.8±9.6 | 19.4± 10.3 |
| **WS [c] (m/s)** | Min | 0.4 | 0.4 | 0.6 | 0.7 | 0.5 | 0.9 |
| | Max | 6.0 | 5.6 | 5.9 | 6.2 | 5.2 | 4.4 |
| | Ave ±SD | 1.8±0.9 | 2.0±0.9 | 2.18±0.9 | 2.1±0.9 | 2.1±0.8 | 2.1±0.6 |
| **RH [d] (%)** | Min | 10.0 | 11.8 | 8.5 | 9.2 | 10.1 | 9.3 |
| | Max | 89.2 | 88.1 | 88.9 | 92.7 | 95.9 | 96.3 |
| | Ave ±SD | 36.1±15.3 | 33.0±11.6 | 35.3±12.7 | 35.4±18.2 | 33.2±15.6 | 33.6±18.9 |
| **Vis [e] (km)** | Min | 2.1 | 2.8 | 2.0 | 1.9 | 0.8 | 1.8 |
| | Max | 13.28 | 13.50 | 12.61 | 12.44 | 12.25 | 11.9 |
| | Ave ±SD | 9.2±1.6 | 9.3±1.5 | 9.3±1.5 | 9.4±1.5 | 9.5±1.3 | 9.5±1.4 |
| **SR ($MJ/m^2$) [e]** | Min | 1.2 | 1.2 | 2.0 | 3.5 | 2.3 | 4.51 |
| | Max | 29.5 | 39.6 | 38.6 | 36.8 | 36.7 | 38.3 |
| | Ave ±SD | 15.7±7.5 | 18.3±3.6 | 21.8±8.4 | 22.1±8.3 | 21.7±4.8 | 26.2±8.8 |
| **Neb [g]** | Min | 1 | 1 | 1 | 1 | 1 | 1 |
| | Max | 8.0 | 7.9 | 8.0 | 7.8 | 8.1 | 8.0 |
| | Ave ±SD | 2.6±2.1 | 2.3±2.3 | 2.6±2.1 | 2.6±2.2 | 2.4±2.1 | 2.2±72.4 |
| **Pre [f] (mm)** | Total | 240 | 158 | 115 | 180 | 165 | 159 |

[a] Meteorological Data; [b] Temperature; [c] Wind Speed; [D] Relative Humidity; [e] Visibility; [f] Precipitation; [e] Solar Radiation; [g] Nebulosity; [h] Minimum; [i] Maximum; [j] Average; [k] Standard Deviation.

**Table S29**. AQI breakpoints suggested by U.S. EPA for criteria air pollutants.

| **AQI subcategories** | 8-hr $O_3$ (ppb) (Lower - Upper) | 1-hr $O_3$ (ppb) (Lower - Upper) | 24-hr $PM_{2.5}$ ($\mu g\ m^{-3}$) (Lower - Upper) | 24-hr $PM_{10}$ ($\mu g\ m^{-3}$) (Lower - Upper) | 8-hr CO (ppm) (Lower - Upper) | 1-hr $SO_2$ (ppb) (Lower - Upper) | 1-hr $NO_2$ (ppb) (Lower - Upper) |
|---|---|---|---|---|---|---|---|
| **Good (< 50)** | 0 - 59 | - | 0.0 - 12.0 | 0 - 54 | 0.0 - 4.4 | 0 - 35 | 0 – 53 |
| **Moderate (51 – 100)** | 60 - 75 | - | 12.1 - 35.4 | 55 - 154 | 4.5 - 9.4 | 36 - 75 | 54-100 |
| **UFSG (101 - 150)** | 76 - 95 | 125 -164 | 35.5 - 55.4 | 155 - 254 | 9.5 - 12.4 | 76 - 185 | 101-360 |
| **Unhealthy (151 – 200)** | 96 - 115 | 165 -204 | 55.5 - 150.4 | 255 - 354 | 12.5 - 15.4 | 186 - 304 | 361-649 |
| **Very unhealthy (201 – 300)** | 116 - 374 | 205 -404 | 150.5 - 250.4 | 355 - 424 | 15.5 - 30.4 | 305 - 604 | 650-1249 |
| **Hazardous (> 300)** | - | 405 -504 | 250.5 - 350.4 | 425 - 504 | 30.5 - 40.4 | 605 - 804 | 1250-1649 |

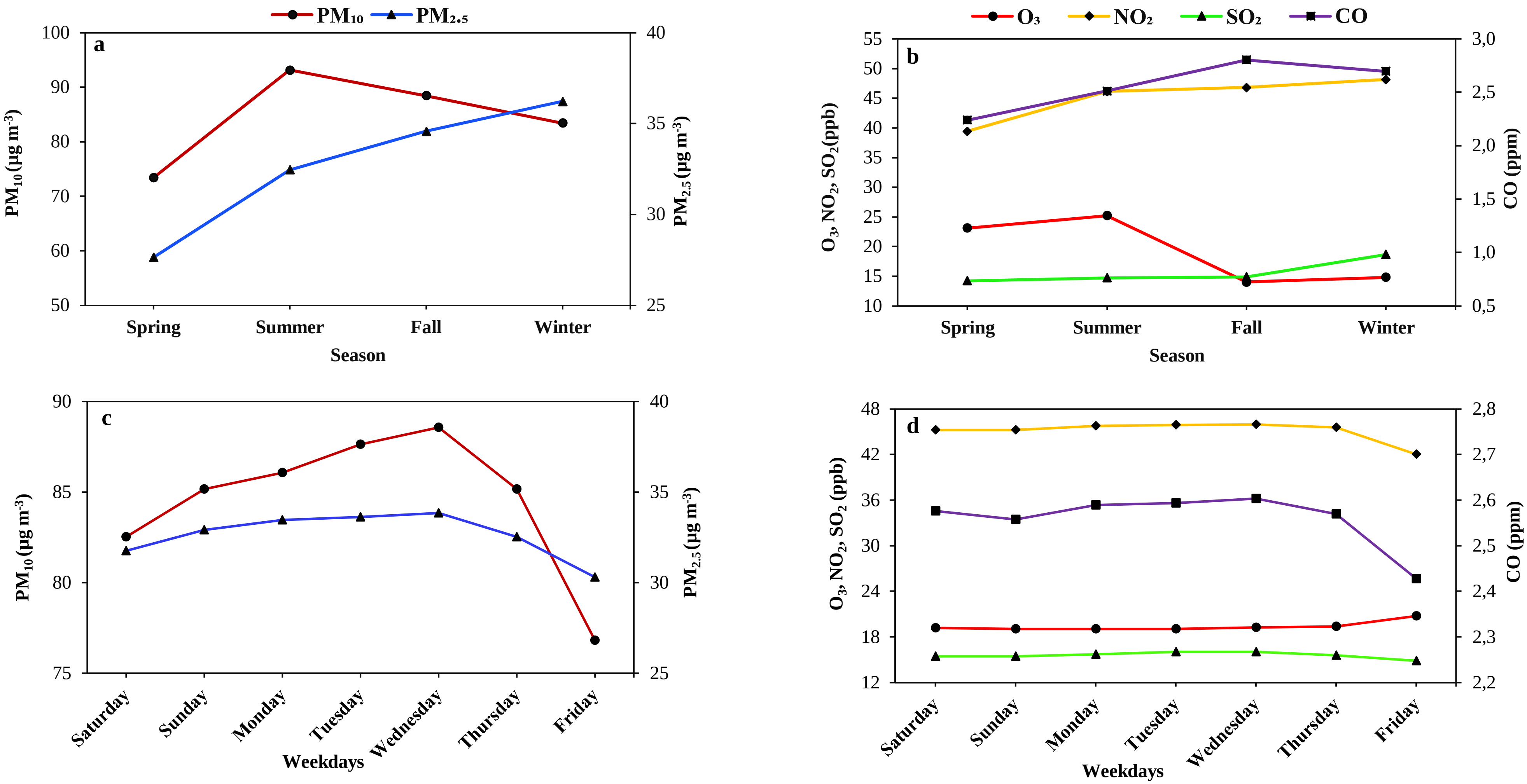


**Figure S1.** Seasonal and daily variations of ambient air pollutants based on hourly concentrations in Tehran during the study period from 2012 to 2017.

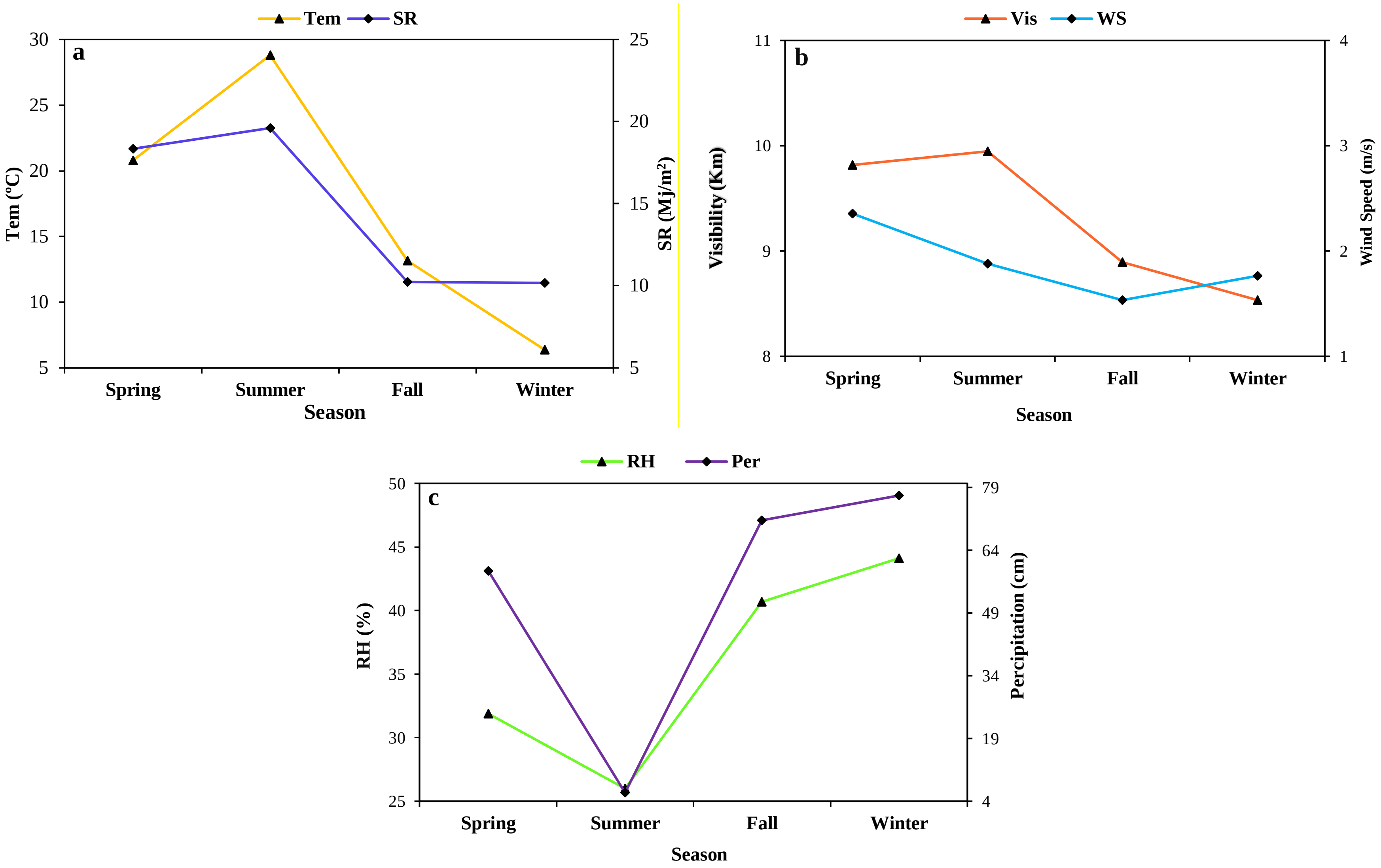


**Figure S2.** Seasonal variation of meteorological parameters (temperature) and SR (solar radiation) (a), WS (wind speed) and visibility (b), RH (relative humidity) and Pre (precipitation) (c) in Tehran during the study period (2012-2017).

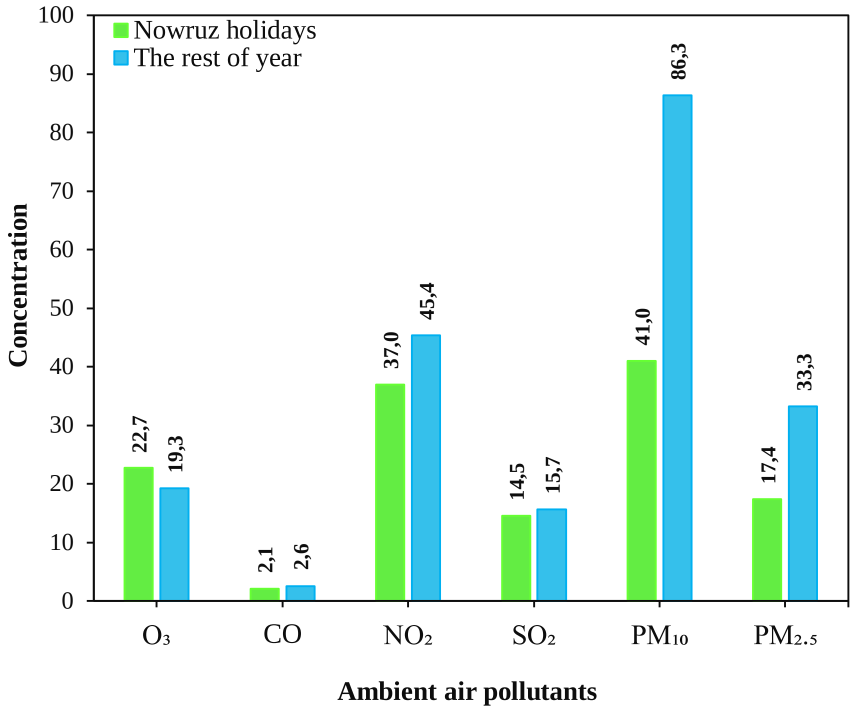


**Figure S3.** The effect of Nowruz holidays on ambient air pollutant concentrations in Tehran during the whole study period (Units: µg $m^{-3}$ for $PM_{2.5}$ and $PM_{10}$, ppb for $O_3$, $NO_2$ and $SO_2$, ppm for CO).

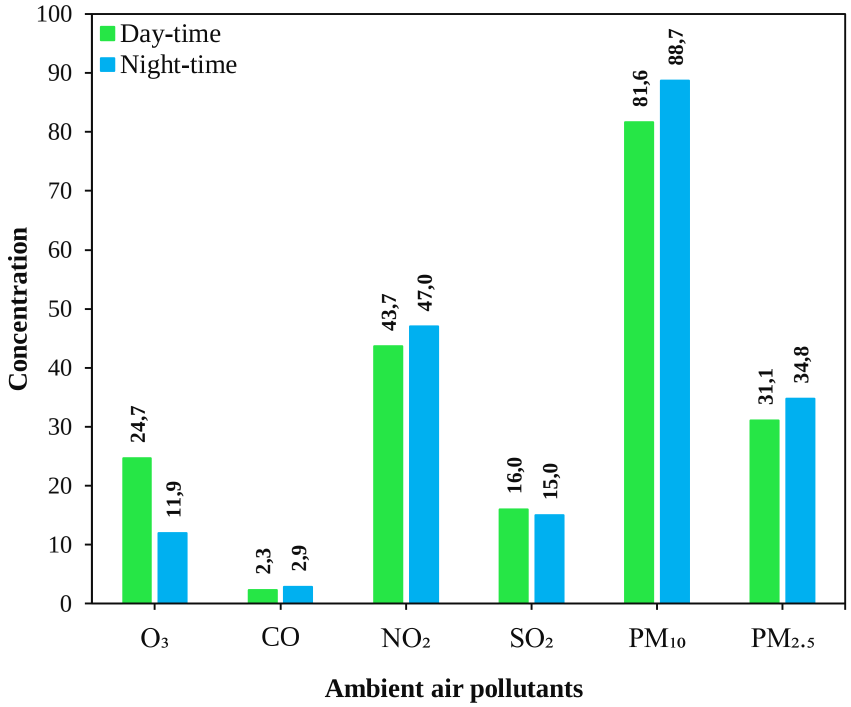


**Figure S4.** Day-time (8:00-20:00) concentrations of ambient air pollutants in comparison to Night-time (21:00-7:00) concentrations in Tehran during the entire study period from 2012 to 2017. (Units: µg $m^{-3}$ for $PM_{2.5}$ and $PM_{10}$, ppb for O3, NO2 and SO2, ppm for CO).

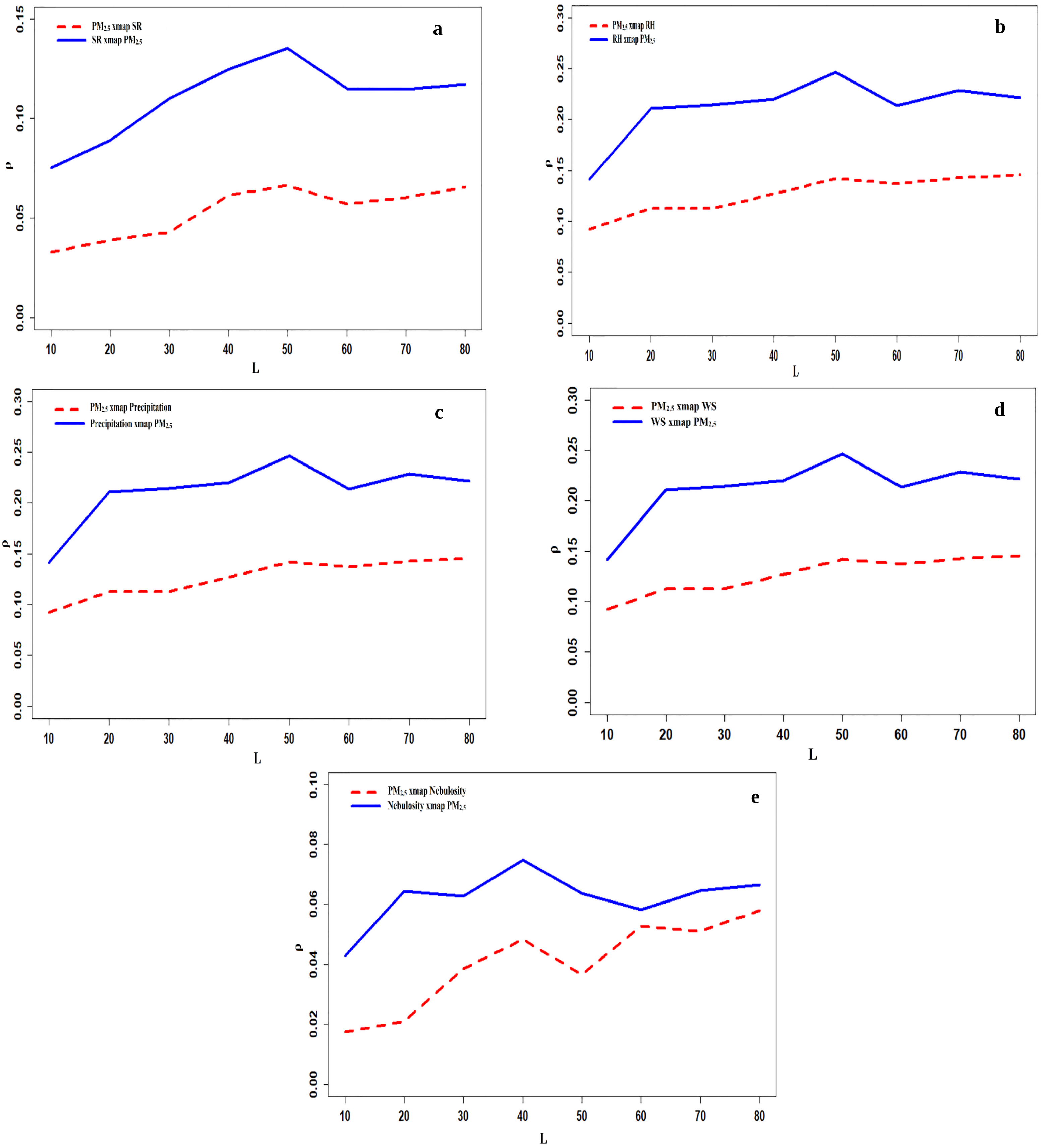


**Figure S5.** CCM test results to show the causality between meteorological parameters and $PM_{2.5}$ concentrations over Tehran, during 2012-2017.

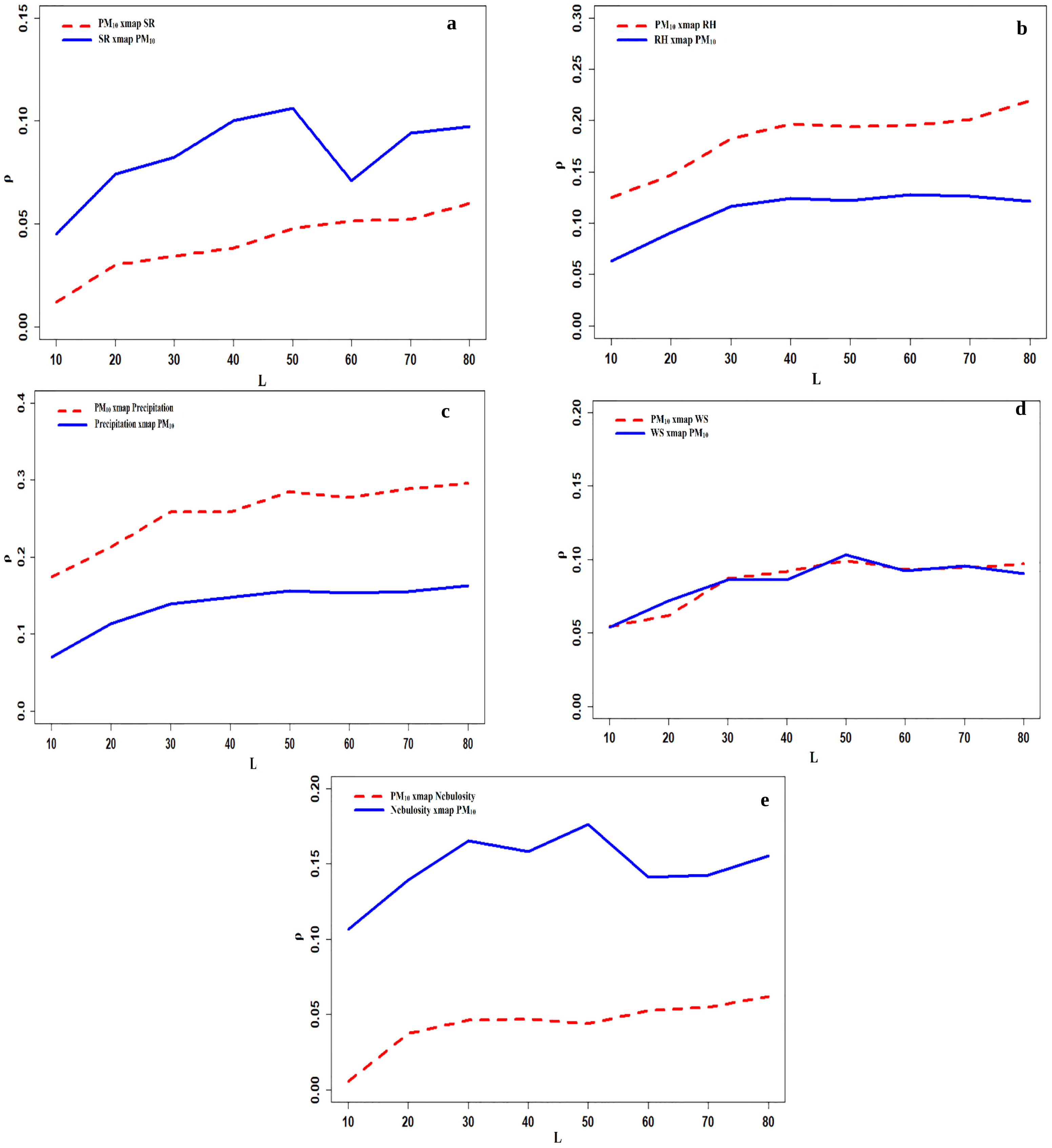


**Figure S6.** CCM test results to show the causality between meteorological parameters and $PM_{10}$ concentrations over Tehran, during 2012-2017.

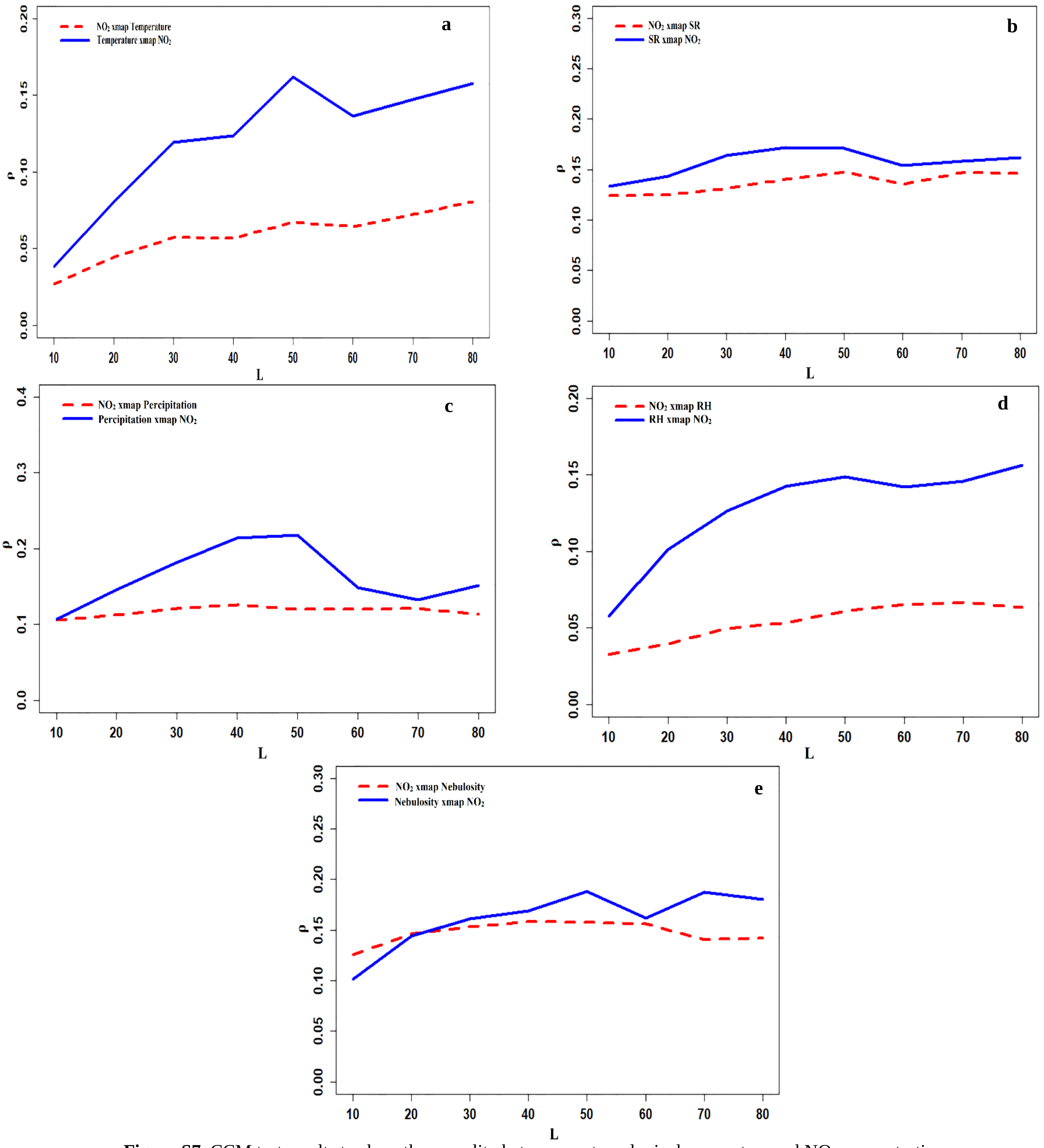


**Figure S7.** CCM test results to show the causality between meteorological parameters and $NO_2$ concentrations over Tehran, during 2012-2017.

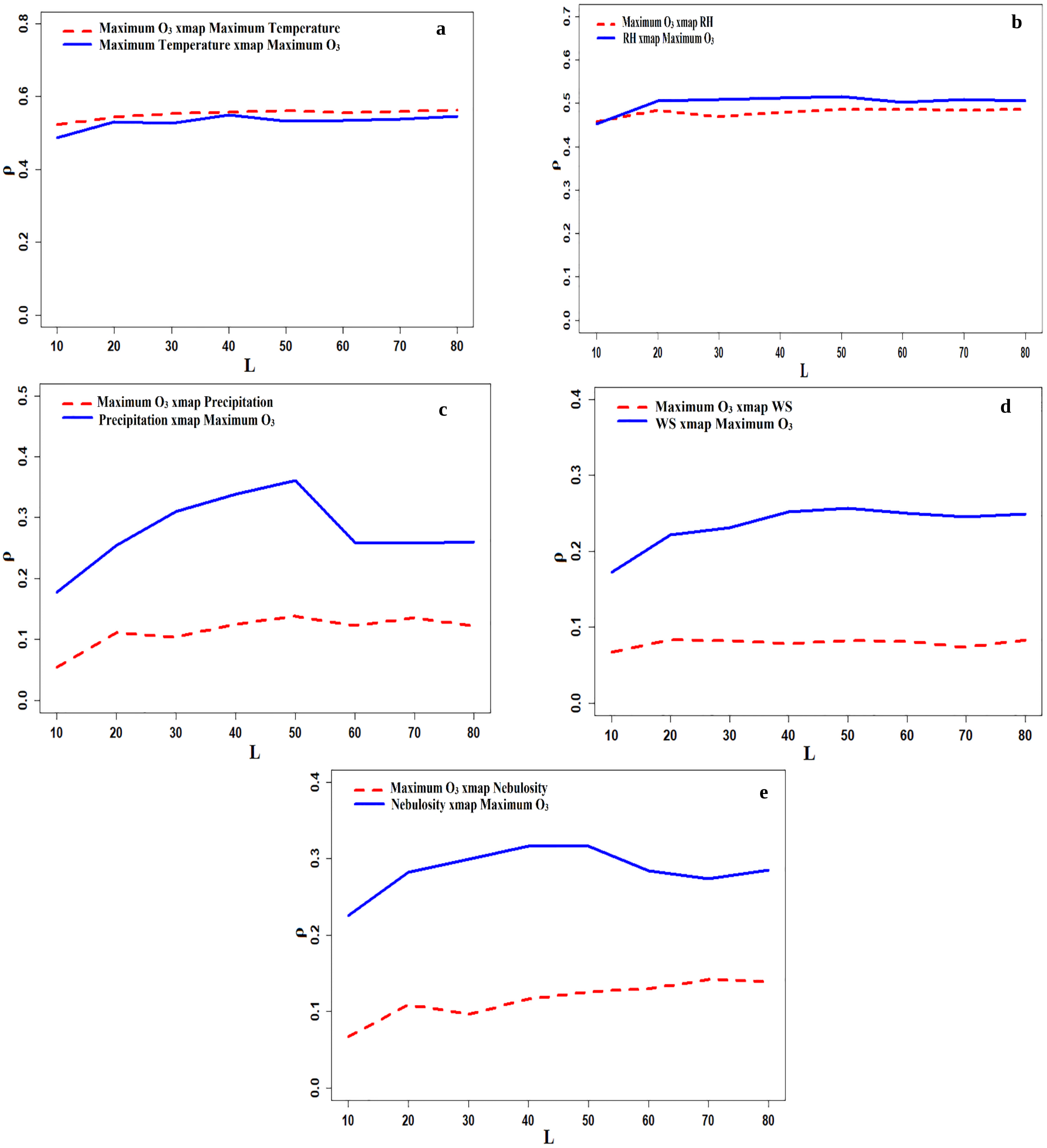


**Figure S8.** CCM test results to show the causality between meteorological parameters and $O_3$ concentrations over Tehran, during 2012-2017.

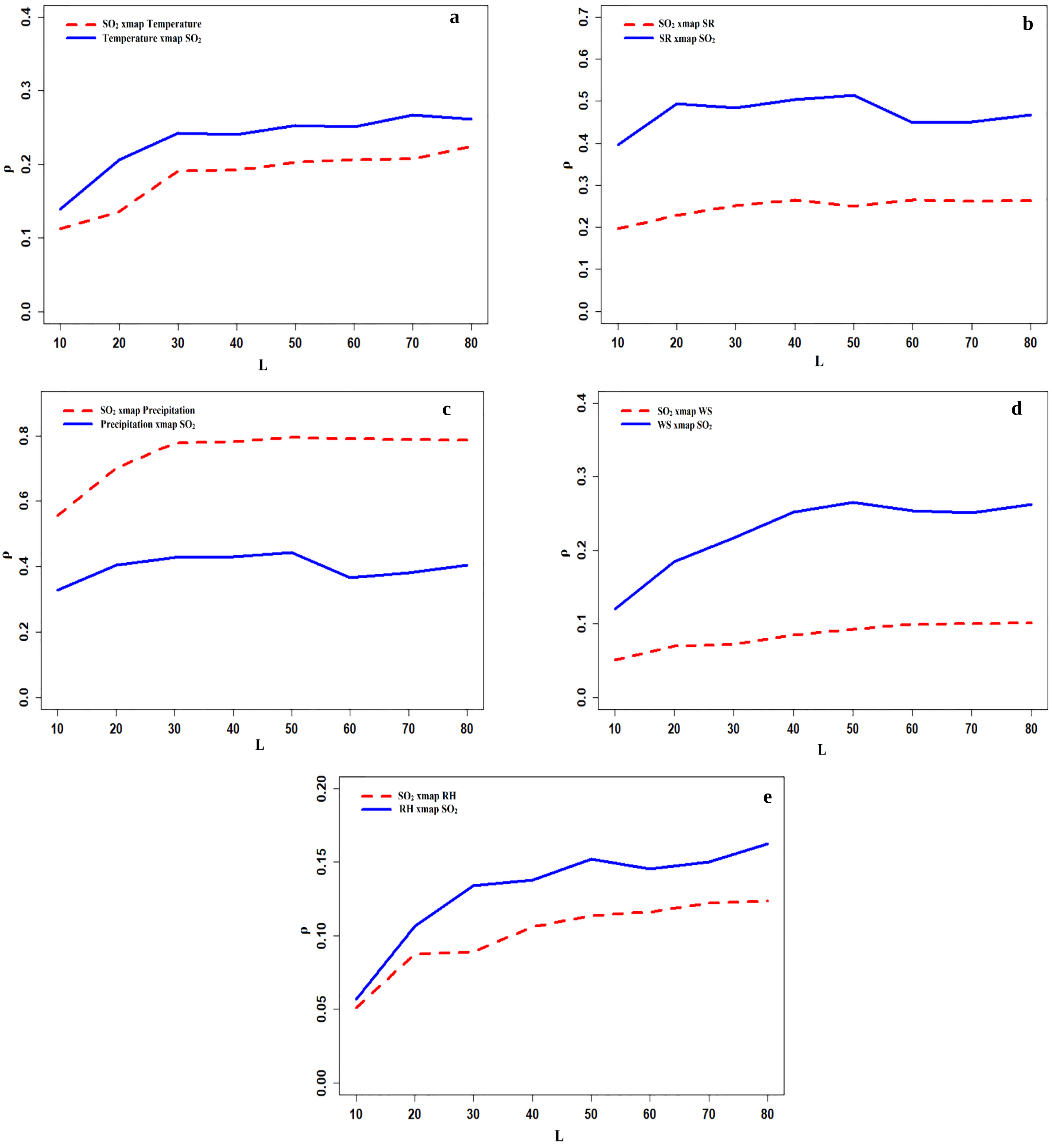


**Figure S9.** CCM test results to show the causality between meteorological parameters and $SO_2$ concentrations over Tehran, during 2012-2017.

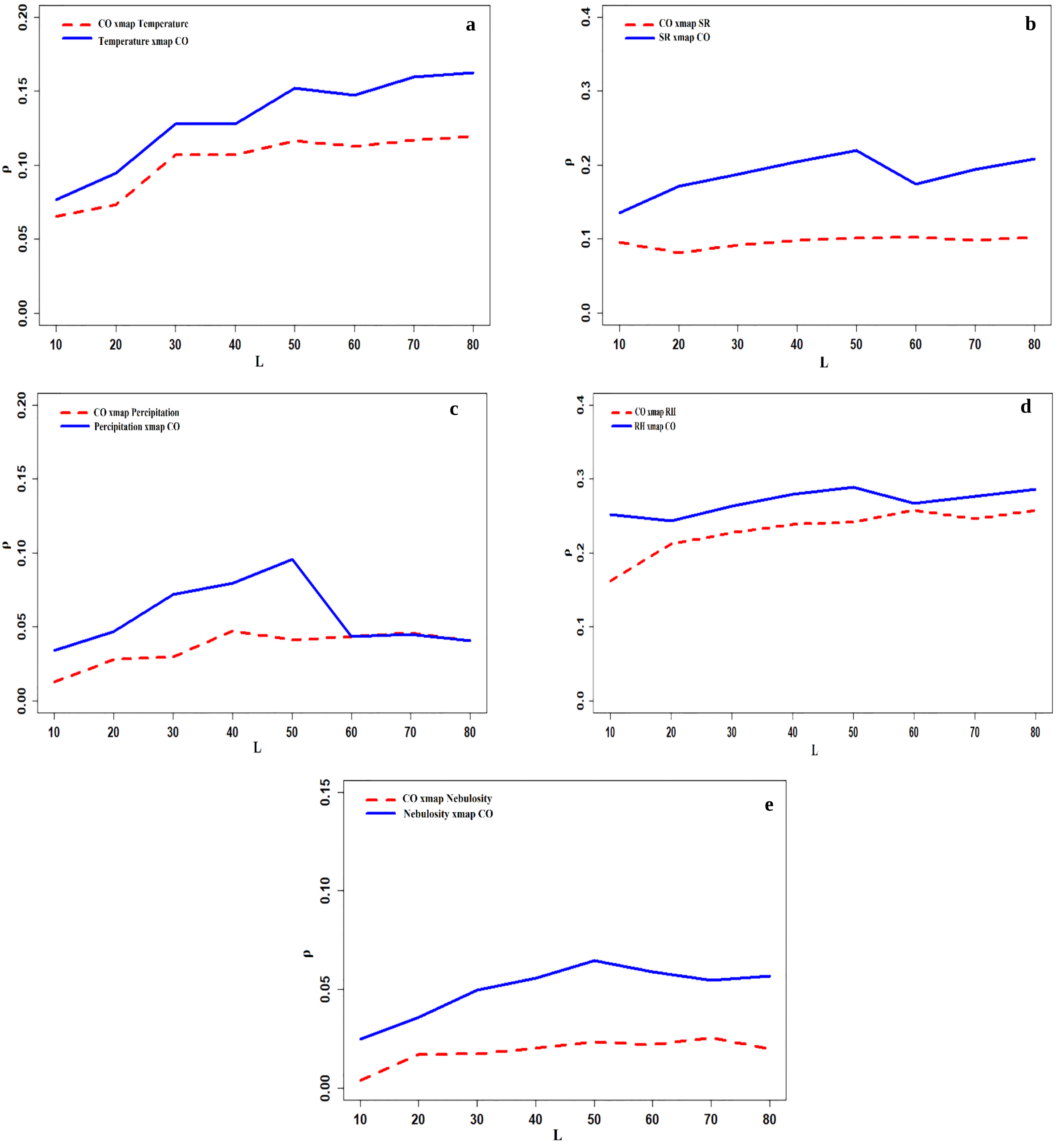


**Figure S10.** CCM test results to show the causality between meteorological parameters and $SO_2$ concentrations over Tehran, during 2012-2017.

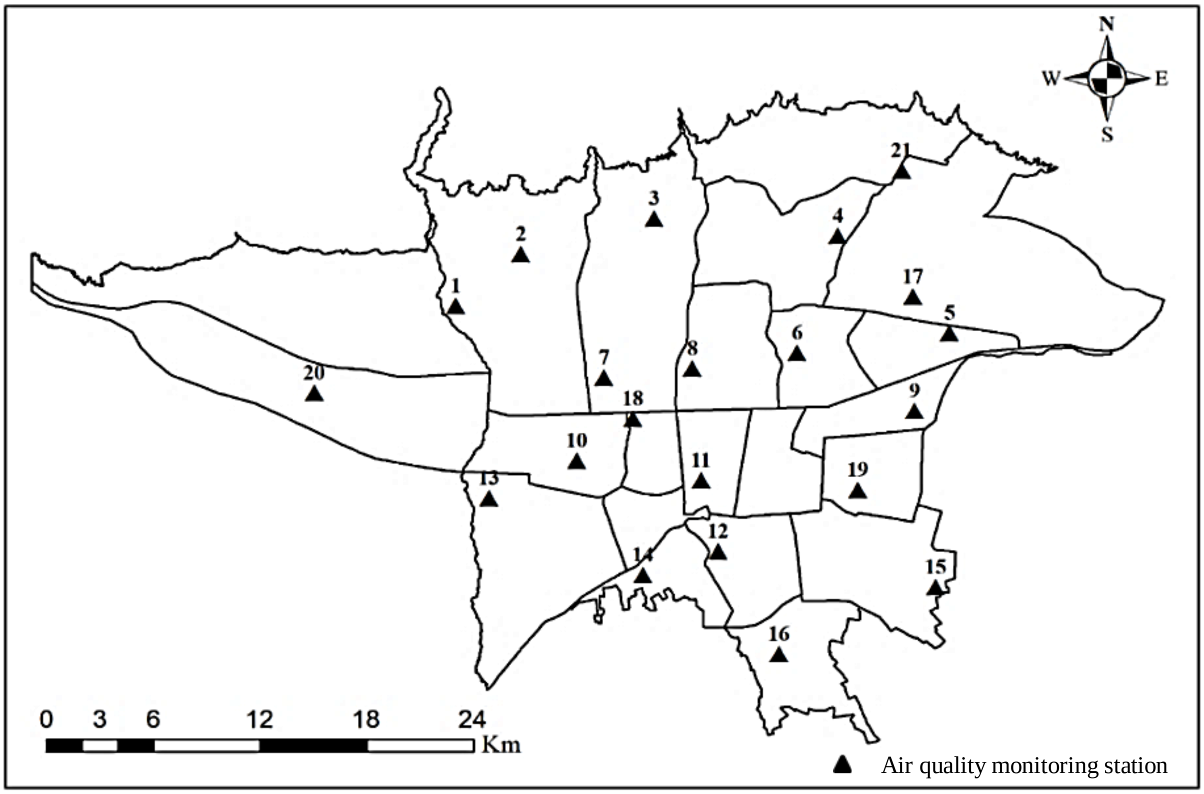


**Figure S11.** The spatial distribution of Air Quality Monitoring Stations (AQMSs) in Tehran.
